# The rhizosphere microbiome plays a role in the resistance to soil-borne pathogens and nutrient uptake of strawberry cultivars under field conditions

**DOI:** 10.1038/s41598-021-82768-2

**Published:** 2021-02-04

**Authors:** Cristina Lazcano, Eric Boyd, Gerald Holmes, Shashika Hewavitharana, Alexis Pasulka, Kelly Ivors

**Affiliations:** 1 Department of Land, Air and Water Resources, University of California Davis, One Shields Avenue, Davis, CA 95616-8627 USA; 2 Department of Natural Resources Management and Environmental Sciences, California Polytechnic State University, San Luis Obispo, CA 93407 USA; 3 Strawberry Center, California Polytechnic State University, San Luis Obispo, CA 93407 USA; 4 Biological Sciences Department, California Polytechnic State University, San Luis Obispo, CA 93407 USA

**Keywords:** Agroecology, Applied microbiology, Microbial ecology, Microbiome

## Abstract

Microbial-root associations are important to help plants cope with abiotic and biotic stressors. Managing these interactions offers an opportunity for improving the efficiency and sustainability of agricultural production. By characterizing the bacterial and archaeal community (via 16S rRNA sequencing) associated with bulk and rhizosphere soil of sixteen strawberry cultivars in two controlled field studies, we explored the relationships between the soil microbiome and plant resistance to two soil-borne fungal pathogens (*Verticillium dahliae* and *Macrophomina phaseolina*). Overall, the plants had a distinctive and genotype-dependent rhizosphere microbiome with higher abundances of known beneficial bacteria such as Pseudomonads and *Rhizobium*. The rhizosphere microbiome played a significant role in the resistance to the two soil-borne pathogens as shown by the differences in microbiome between high and low resistance cultivars. Resistant cultivars were characterized by higher abundances of known biocontrol microorganisms including actinobacteria (*Arthrobacter*, *Nocardioides* and *Gaiella*) and unclassified acidobacteria (Gp6, Gp16 and Gp4), in both pathogen trials. Additionally, cultivars that were resistant to *V. dahliae* had higher rhizosphere abundances of *Burkholderia* and cultivars resistant to *M. phaseolina* had higher abundances of *Pseudomonas*. The mechanisms involved in these beneficial plant-microbial interactions and their plasticity in different environments should be studied further for the design of low-input disease management strategies.

## Introduction

Soil microorganisms support key soil processes such as decomposition, mineralization, aggregate formation and biocontrol of plant diseases, all highly relevant to agricultural production^1–3^. However, understanding the complexity and drivers of the soil microbiome, remains a challenge, and the practical application of ecologically based agricultural management strategies not feasible^4^. Soil bacterial diversity and abundance is primarily regulated by small- and large-scale changes in abiotic factors such as soil pH, oxygen concentration, and the availability as well as quality of carbon (C)^1^. Large variations in these factors exist in the macro (i.e. cm scale) and microenvironments (i.e. mm to μm scale) within a soil profile, producing hotspots of microbial diversity and activity. The rhizosphere, the volume of soil that is directly influenced by plant roots and which spans usually 1 mm from the root surface, can contain up to 30,000 bacterial and archaeal species^5^. Furthermore, the rhizosphere typically has higher microbial biomass and activity when compared to the surrounding bulk soil^6^. This microbial hotspot within soil habitats is driven by plant root presence, nutrient and water uptake as well as the continuous release of carbon compounds through root rhizodeposits^7,8^. Differential bioavailability and assimilation of carbon compounds by soil microorganisms results in the selective promotion of certain taxa and inhibition of others^9^.

Root-associated microorganisms, either symbiotic or free living are key for plant nutrition and health and, because of this, the rhizosphere is frequently compared with the human gut^10^. Soil bacteria such as *Pseudomonas*, *Bacillus* and *Rhizobium* can increase the concentration of plant available phosphorus (P) in soil by releasing phosphatase enzymes or organic chelates^11^. Root-exudate production and the release of C compounds to usually C-deprived soil microorganisms, is known to trigger microbial activity and the production of exoenzymes which in turn further accelerates the decomposition of organic molecules in the rhizosphere and nutrient release^12,13^. As a result, rhizosphere soil typically has larger N mineralization rates and higher concentrations of bioavailable nitrogen (N) and P when compared to bulk soil, regardless of the plant species considered^6^. Additionally, certain strains of *Bacillus*, *Pseudomonas, Burkholderia* and *Acinetobacter* are thought to increase the availability and uptake of micronutrients such as Zn and Fe for mung bean, rice and corn^14–16^. Increases in plant growth have also been related to the induction of changes in plant root architecture and increase in root absorptive surface through the bacterially secreted plant hormones like indole-3-acetic acid, gibberellins and cytokinins^17^. Plants with adequate nutrition may be more resilient to biotic stress; in addition, rhizosphere microorganisms are known to have also direct impacts in plant health by triggering plant defenses^10^. Induction of systemic resistance in tomato plants by the bacterium *Azospirillum brasilense* was attributed to the capacity of this microbial strain to produce ethylene, a hormone involved in plant defense^17^. In tomato plants rhizosphere, bacteria from *Bacillus, Pseudomonas*, and *Azotobacter* triggered the production of antioxidant peroxidase and polyphenol oxidase enzymes, reducing the incidence of early blight disease by the pathogenic fungus *Alternaria solani*^18^. Finally, numerous bacteria have been identified that are antagonists of filamentous fungal pathogens such as *Verticillium dahliae* and are therefore able to directly suppress the pathogen via competition, production of antibiotic substances, siderophores, protein degrading enzymes and volatile compounds^19^.

Cultivated strawberry (*Fragaria x ananassa* Duchesne ex Rozier) is a fruit crop highly appreciated throughout the world, and therefore of major economic importance^20^. World production of this crop increased sharply over the last decades reaching over 13 million tons in 2017^21^. Currently, the largest producer is China, followed by the USA, Mexico, Egypt, Turkey and Spain^21^. Within the USA, California strawberry production accounted for 88% of the U.S. market in 2017, representing the 4th most valuable agricultural commodity in the state with a total revenue of 3.10 billion dollars^22^. Since the late 1950s, the California strawberry industry relied on the use of preplant soil fumigation with methyl bromide to control soil-borne diseases^23^. However, since the complete phase out of methyl bromide in 2016 under the Montreal Protocol due to its stratospheric ozone depleting nature, strawberry growers are facing the challenge of maintaining their productivity while using alternative disease management strategies^24^. At the same time, new diseases such as the soil-borne fungus *Macrophomina phaseolina*, are starting to emerge threatening the strawberry industry even further^25^. Other fumigants such as chloropicrin are currently in use, although their efficacy is substantially lower than methyl bromide^26^. Furthermore, urban development and encroachment in the areas with the most intensive strawberry production, severely limits the application of pesticides. The above-mentioned restrictions in fumigant use have shifted the interest of the industry towards integrated strategies combining cultural techniques such as anaerobic soil disinfestation, cover crops, or crop rotations to improve soil health, as well as the use of resistant cultivars^23^. To date, strawberry cultivars have been identified that are tolerant to soil-borne pathogens like *Verticillium dahliae*^27,28^ and *Fusarium oxysporum* f. sp. *fragariae*. Resistance to these soil-borne pathogens seems to be achieved through the expression of multiple genes^29^ thought to regulate the production of substances with antifungal properties such as catechin, caffeic acid, or citric acid^30^. Cultivars with different degrees of resistance to Macrophomina crown rot have also been identified, yet little is known on the mechanisms involved despite its recently increasing incidence in California and other regions of the world^31–33^. The interplay between roots and soil microorganisms is likely involved in the resistance to these soil-borne fungal pathogens, as it has been previously observed for cucumber, common bean and tomato^34–36^. Yet, to date no information is available on how the rhizosphere microbiome influences the resistance of strawberry cultivars to these pathogens. This knowledge is instrumental in the selection of strawberry cultivars that will allow the strawberry industry to maintain its high yields and competitiveness, while reducing the need for agrochemicals with the associated reduction in production costs and environmental impacts.

Here, we used two ongoing field trials established to determine the host resistance to *M. phaseolina* and *V. dahliae* of strawberry cultivars and elite breeding lines, to explore potential resistance mechanisms by assessing the role of the rhizosphere microbiome in the resistance to these soil-borne fungal pathogens in a subset of 16 cultivars. More precisely we aimed to: (i) characterize the rhizosphere microbiome in strawberry plants under commercial field conditions; (ii) identify changes in the rhizosphere microbiome in the presence of two soil-borne fungal pathogens (*V. dahliae* and *M. phaseolina*) and potential increases in the presence of beneficial rhizosphere microorganisms and (iii) evaluate the relationships between the rhizosphere microbiome and plant traits such as resistance against soil-borne pathogens, plant growth and nutrition. We hypothesized that strawberry plants have the capacity to recruit beneficial microorganisms and establish a rhizosphere microbiome different from bulk soil. Further, variations in plant traits derived from decades of breeding cultivars would also have an impact on belowground root traits and therefore different strawberry cultivars would show different microbial communities. Finally, plants with higher resistance will exhibit a different microbiome from plants with low resistance having a higher abundance of beneficial bacteria and archaea; resistance to soil-borne pathogens, growth and nutrient uptake, would be corelated with the composition of the rhizosphere microbiome.

## Results

### Rhizosphere microbiome of the strawberry cultivars

A total of 3,939,353 high quality bacterial and archaeal sequences were generated across both the bulk soil and rhizosphere soil samples. After filtering chloroplast and unassigned reads, 3,786,463 sequences were clustered into 5,600 OTUs representing 33 Archaeal and 5567 Bacterial OTUs from 353 genera.

**Figure 1:**
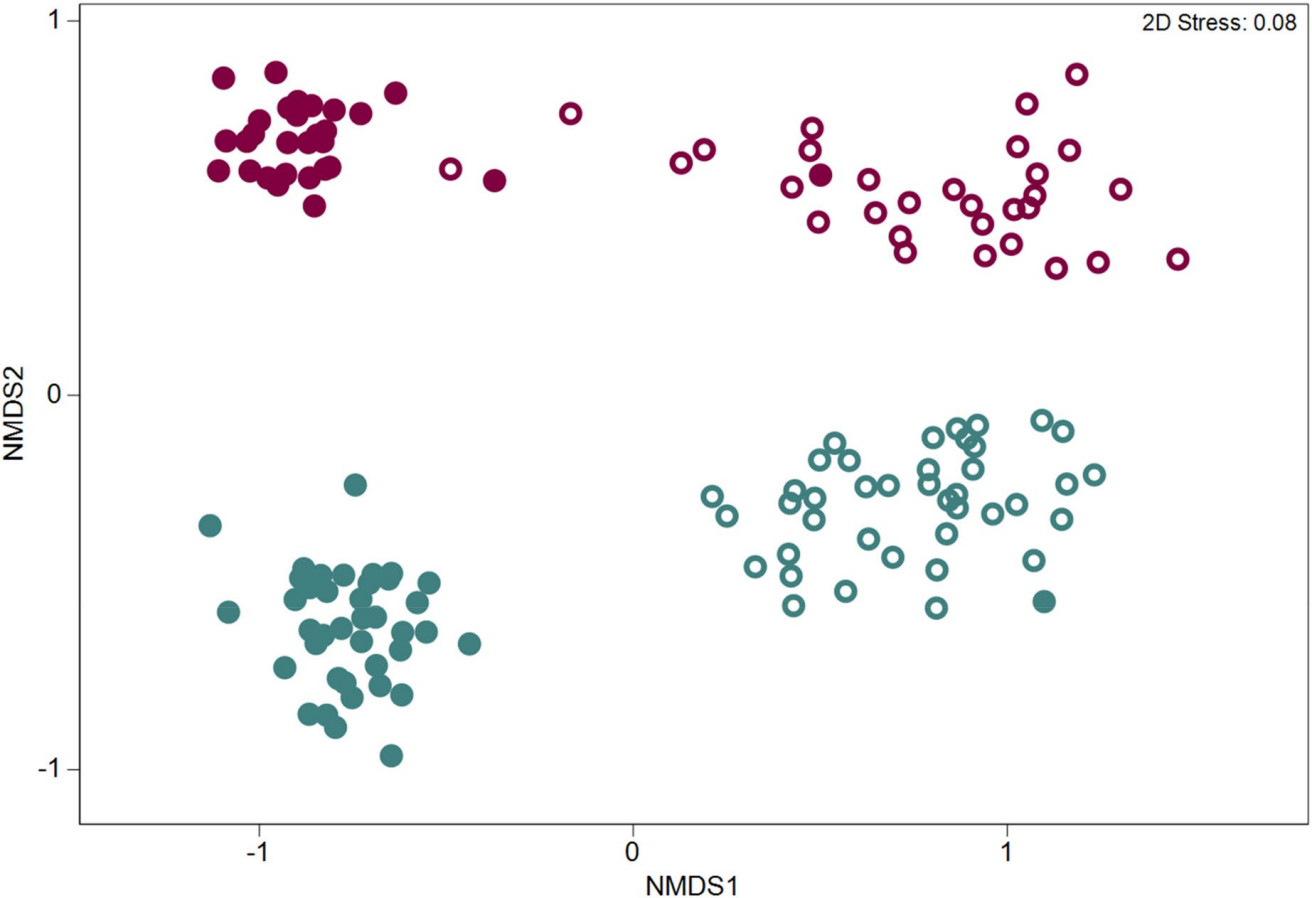
Non-metric multidimensional scaling (NMDS) ordinations based on the Bray–Curtis similarity of the OTU-based bacterial and archaeal community structure in the rhizosphere (open symbols) and bulk soil (filled symbols) in two pathogen trials inoculated or infested with *Macrophomina phaseolina* (blue) and *Verticillium dahliae* (red).

Differences in OTU community structure between sample types (rhizosphere and bulk soils) in the two pathogen trials were examined via non-metric multidimensional scaling (NMDS) using a Bray–Curtis dissimilarity of the standardized and transformed data (Fig. 1).

We observed significant differences in the soil microbiome between pathogen trials although the magnitude of the differences depended on the sample type (PERMANOVA results for pathogen trial x sample type: Pseudo-F = 9.17, *p* = 0.001) (Fig. 1). A pairwise comparison via PERMANOVA between rhizosphere soils in each pathogen trial showed significant differences in their microbiomes (t = 4.14, *p* = 0.001).

**Figure 2:**
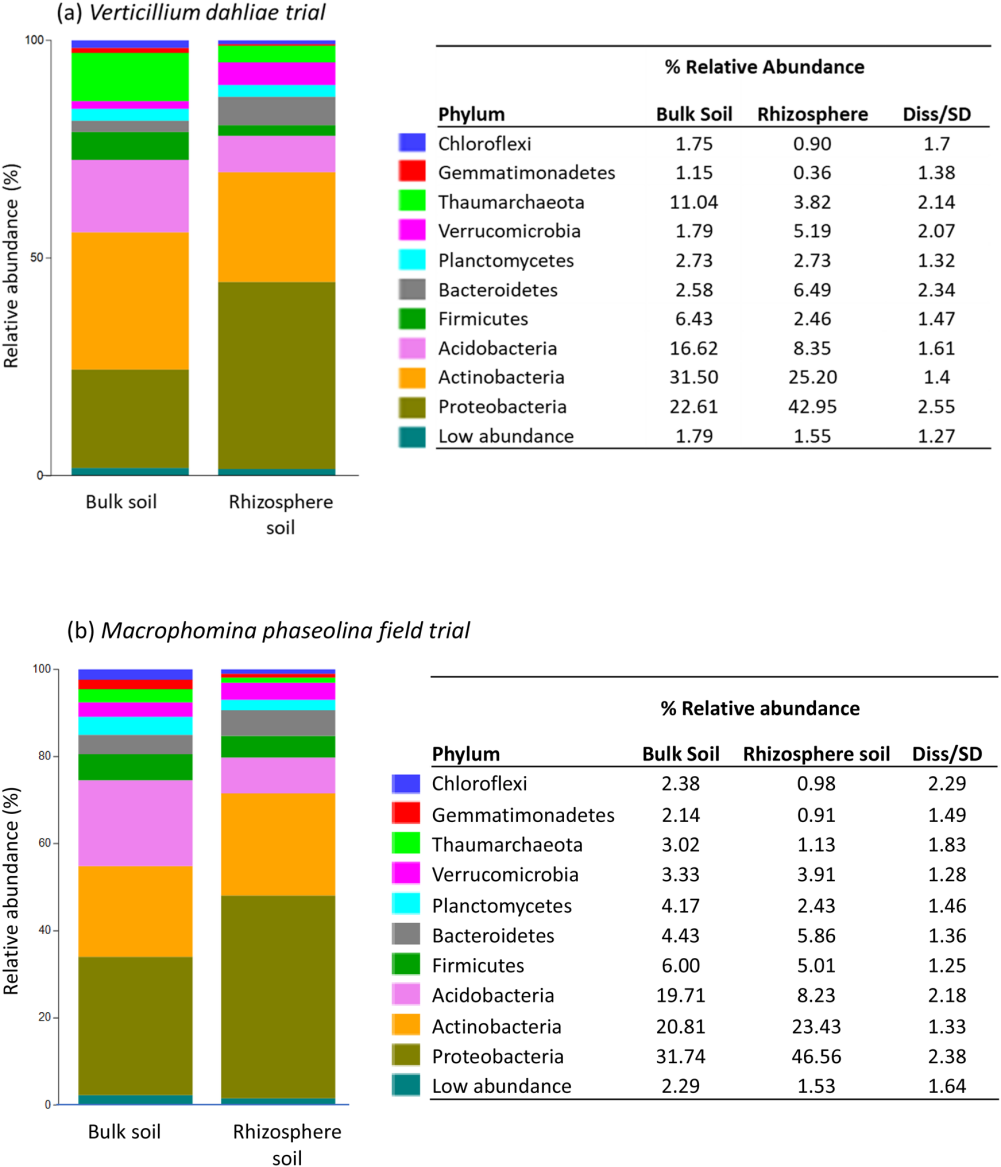
Differences in the average relative abundance of the main archaeal and bacterial phyla in the bulk and rhizosphere soil samples collected from the pathogen trials infested with *Verticillium dahliae* (**a**) and inoculated with *Macrophomina phaseolina* (**b**). Phyla representing < 1% of the average relative abundance were grouped into ‘low abundance’.

SIMPER analysis (performed with taxa grouped at the phylum level) revealed that the rhizosphere and bulk soil samples differed by 17.87% and 13.56% in the *V. dahliae* and *M. phaseolina* trial, respectively. In the *V. dahliae* trial, the bacterial phyla contributing most to these differences included the archaeal phylum Thaumarchaeota, Firmicutes and Acidobacteria, which exhibited higher abundances in the bulk soil relative to the rhizosphere soil (Fig. 2a). Additionally, the phyla Verrucomicrobia, Bacteroidetes, and Proteobacteria exhibited higher abundances in the rhizosphere soil relative the bulk soil (Fig. 2a). In the *M. phaseolina* trial, the archaeal phylum Acidobacteria contributed the most to differences between sample types, followed by the archaeal phylum Thaumarchaeota and the bacterial phyla Chloroflexi, Gemmatimonadetes, Planctomycetes and Firmicutes, all of which exhibited higher abundances in the bulk soil relative to rhizosphere soil (Fig. 2b). The phyla Proteobacteria, Verrucomicrobia, Bacteroidetes and Actinobacteria exhibited a greater relative abundance in the rhizosphere soil compared with the bulk soil (Fig. 2b).

**Figure 3:**
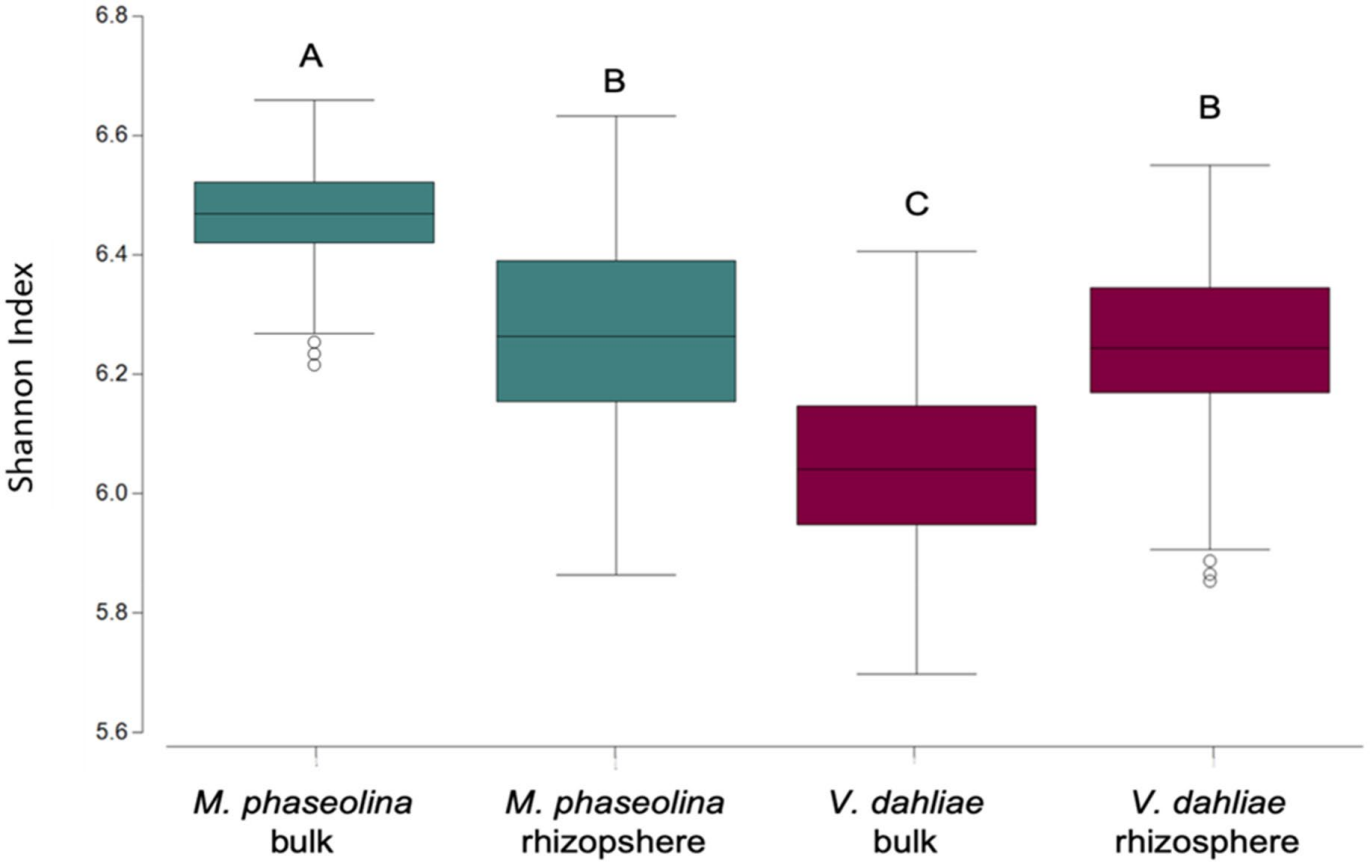
Diversity of the microbial community in the bulk and rhizosphere soil from the pathogen trials inoculated with *Macrophomina phaseolina* and infested with *Verticillium dahliae*. Different letters denote significant differences at *p* < 0.005.

Diversity of OTUs, determined using the Shannon diversity index (H) was significantly higher in the bulk soil of the *M. phaseolina* trial than in the *V. dahliae* trial but remained similar in the rhizosphere soil across trials (pathogen trial x sample type: F = 40.75, *p* < 0.0001; Fig. 3).

**Figure 4:**
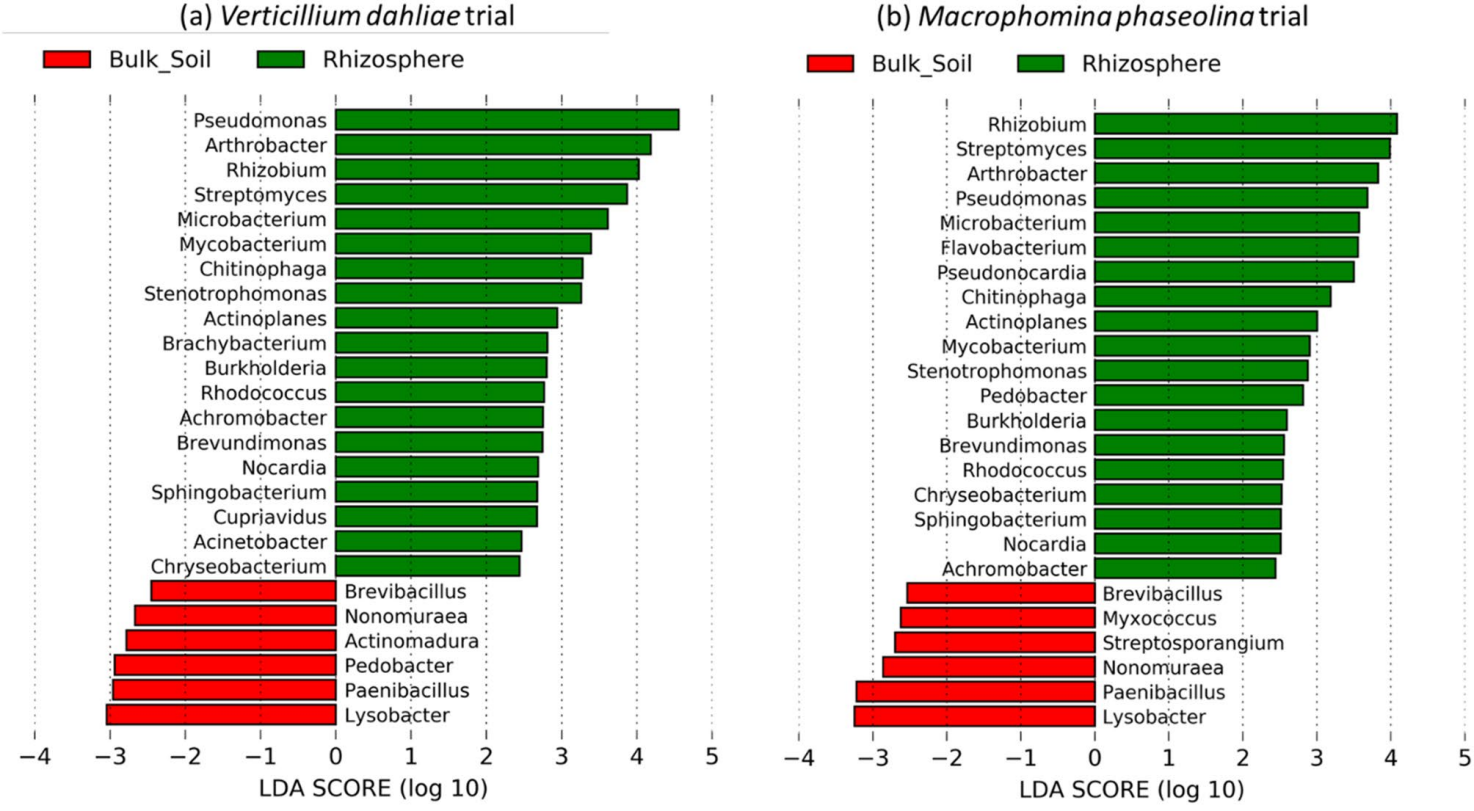
Linear discriminant analysis (LDA) effect size (LEfSe) of genera known to include species antagonistic to filamentous fungal pathogen in the rhizosphere and bulk soil of strawberry grown in fields infested with *Verticilium dahliae* and inoculated with *Macrophomina phaseolina*. Genera listed were both significantly different (α = 0.05) and had an LEfSe score of > 2.0. A positive LDA score represents depletion in bulk soil and enrichment in rhizosphere (green) and a negative LDA score represents the opposite (red).

We used linear discriminant effect size analysis (LEfSe) to assess the differences in the relative abundance of the different microorganisms at the genus level, specifically focusing on a subset of well-known antagonists of filamentous fungal plant pathogens^19^. The LEfSe analysis performed on samples from within in the *V. dahliae* trial data revealed 191 taxonomic clades that exhibited greater relative abundance in the bulk soil relative to the rhizosphere (Table S1), 6 of which are known fungal antagonists (Fig. 4a). This analysis also revealed 231 taxonomic clades that exhibited greater relative abundances in the rhizosphere, 19 of which are genera known to include fungal antagonistic species (Fig. 4a). On samples from within the *M. phaseolina* trial, LefSe analysis revealed 219 taxonomic clades that exhibited greater relative abundance in the bulk soil relative to the rhizosphere soil, 6 of which are known fungal antagonists (Table S1). Additionally, there were 220 taxonomic clades that exhibited greater relative abundances in the rhizosphere, 19 of which are known fungal antagonists (Fig. 4b).

### Effects of the soil-borne fungal pathogens on the rhizosphere microbiome

The effect of pathogen presence on the rhizosphere microbiome of strawberry plants was determined by comparing the rhizosphere microbiomes in the non-infested controls with the experimental plots that were inoculated (*M. phaseolina* trial) or grown in soil with a high concentration of the fungal pathogen (*V. dahliae* trial). To compare control vs inoculated plants, we aggregated all the plots, regardless of the cultivar. Thus, the control had a total of 10 replicates (plots), while inoculated plants had 40 replicates (plots) in each trial. The rhizosphere microbial community composition of plants exposed to *V. dahliae* was significantly different from control plants (ANOSIM: R = 0.3, *p* = 0.004). An average dissimilarity of 27.11% in OTU relative abundances was found between infested and control plants based on SIMPER analysis (performed with taxa grouped at the genus level) in the *V. dahliae* trial. The genera found in higher relative abundance in the control plants include *Arthrobacter*, *Steroidobacter*, *Pseudomonas*, *Azotobacter*, *Luteolibacter*, *Nakamutella*, Saccharibacteria (genera incertae sedis), WPS-1 (genera incertae sedis), *Micromonospora*, *Arenimonas*. On the other hand, the archaeal genus Nitrosphaera and bacterial genera *Gaiella*, *Hydrocarboniphaga*, *Rubrobacter*, *Streptomyces*, *Mucilaginibacter*, *Rhizobium*, *Pseudonocardia*, *Niastella* and *Aminobacter* exhibited greater relative abundances in *V. dahliae* infested plants (Table 1).

**Table 1:** Average percent relative abundances of bacterial genera contributing to at least 1% to the observed variability between the rhizosphere of infested vs. control plants via SIMPER analysis.

Genus	Infested	Control	Diss/SD	Contribution (%)
***Verticilium dahliae field trial***				
*Nitrososphaera*	7.96	2.84	1.43	2.66
*Arthrobacter*	3.26	6.35	1.72	1.99
*Steroidobacter*	2.05	3.55	1.42	1.9
*Gaiella*	4.05	1.73	1.38	1.82
*Pseudomonas*	1.35	2.04	1.23	1.64
*Hydrocarboniphaga*	0.97	0.64	1.25	1.56
*Azotobacter*	0.26	1.43	0.7	1.54
*Rubrobacter*	1.21	1.11	0.88	1.51
*Streptomyces*	3.24	2.87	1.35	1.48
*Mucilaginibacter*	2.61	1.64	1.38	1.44
*Luteolibacter*	0.74	1.52	1.64	1.37
*Nakamurella*	0.55	1.16	1.09	1.33
*Saccharibacteria_genera_incertae_sedis*	0.95	1.8	1.54	1.31
*Rhizobium*	3	2.19	1.32	1.25
*Methylobacterium*	1.1	1.11	1.32	1.25
*WPS-1_genera_incertae_sedis*	1.06	1.74	1.48	1.24
*Micromonospora*	0.16	0.72	2.22	1.22
*Pseudonocardia*	2.36	1.38	1.69	1.14
*Niastella*	0.76	0.59	1.02	1.13
*Aminobacter*	1.24	1.12	1.33	1.09
*Arenimonas*	0.57	0.74	1.33	1.02
***Macrophomina phaseolina field trial***				
*Pseudomonas*	9.1	4.12	1.57	2.49
*Nakamurella*	0.73	2.53	1.48	2.17
*Gemmatimonas*	0.32	1.33	0.92	1.6
*Isoptericola*	0.68	0.63	1.13	1.45
*Arthrobacter*	5.75	6.61	1.35	1.42
*Xanthomonas*	0.89	0.68	1.29	1.39
*Flavobacterium*	1.08	1.69	1.02	1.36
*Phycicoccus*	0.97	1.92	1.41	1.33
*Saccharibacteria genera incertae sedis*	1.18	1.89	1.45	1.29
*Steroidobacter*	2.09	1.59	1.35	1.19
*Streptomyces*	2.34	1.62	1.38	1.16
*Burkholderia*	0.15	0.58	1.07	1.16
*Mucilaginibacter*	1.64	0.91	1.36	1.13
*Bradyrhizobium*	0.74	1.06	1.27	1.09
*Gaiella*	1.78	1.16	1.43	1.09
*Novosphingobium*	4.2	4.38	1.34	1.07
*Nitrososphaera*	2.46	1.77	1.45	1.07
*Luteolibacter*	0.67	0.92	1.35	1.06
*Massilia*	0.67	0.53	1.2	1.03
*Arenimonas*	0.55	0.81	1.46	1.02
*Sphingomonas*	4.32	5.26	1.37	1.01

Diss/SD values represent the average dissimilarity divided by the standard deviation.

The rhizosphere microbiome was also significantly different between inoculated and control plants in the *M. phaseolina* trial (R = 0.51, *p* < 0.001). SIMPER analysis (performed with taxa grouped at the genus level) revealed an average dissimilarity of 27.07% between infested and non-infested treatments. The bacterial genera *Nakamurella*, *Gemmatimonas*, *Arthrobacter*, *Flavobacterium*, *Phycicoccus*, *Saccharibacteria* (*genera incertae sedis*), *Burkholderia*, *Bradyrhizobium*, *Novosphingobium*, *Luteolibacter*, *Arenimonas* and *Sphingobium* exhibited higher relative abundances in the control relative to the inoculated plants and contributed at least 1% to the total dissimilarity between groups. The bacteria in the genera *Pseudomonas*, *Isoptericola*, *Xanthomonas*, *Steroidobacter*, *Streptomyces*, *Mucilaginibacter*, *Gaiella* and *Massilia* exhibited higher relative abundances in the rhizosphere of plants inoculated with *M. phaseolina* and contributed at least 1% to the total dissimilarity between groups (Table 1).

### Cultivar-dependent selection of rhizosphere microorganisms

Within the fungal-inoculated plots, significant differences were observed in the rhizosphere microbiome of the 10 strawberry cultivars grown in the *V. dahliae* trial (ANOSIMS R = 0.43, *p* < 0.001), showing the cultivar-dependent selection of microorganisms. Similar results were observed for the 10 cultivars grown in the *M. phaseolina* trial (ANOSIMS R = 0.24, *p* < 0.001). Furthermore, Shannon diversity in the rhizosphere microbiome was also found to be significantly different between cultivars in the *V. dahliae* trial (ANOVA: F = 2.41, *p* = 0.05, Table 2), but not in the *M. phaseolina* trial (ANOVA: F = 0.58, *p* = 0.8, Table 3).

**Table 2:** Aboveground traits (biomass and leaf nutrient contents), rhizosphere microbial diversity (Shannon Index) and mortality of the strawberry cultivars grown in the presence of the soil-borne fungal pathogen *Verticillium dahliae*.

	Mortality (%)	Resistance	Plant biomass (g)	Leaf N (%)	Leaf P (%)	Leaf K (%)	Leaf Ca (%)	Leaf Mg (%)	Leaf Ca:Mg	Shannon Index
UC	2.2 ± 2.2^c^	High	96.44 ± 5.64^abc^	1.79 ± 0.09^bc^	0.30 ± 0.01^a^	1.16 ± 0.06^ab^	1.20 ± 0.12^ab^	0.52 ± 0.03^ab^	2.29 ± 0.10^ab^	6.17 ± 0.16
CR	4.5 ± 2.2^c^	High	60.95 ± 11.25^cde^	1.94 ± 0.08^bc^	0.33 ± 0.01^a^	1.00 ± 0.04^b^	1.26 ± 0.08^ab^	0.54 ± 0.01^ab^	2.32 ± 0.11^ab^	6.25 ± 0.02
MS	4.5 ± 2.2^c^	High	117.32 ± 11.08^a^	1.80 ± 0.05^bc^	0.28 ± 0.01^ab^	1.24 ± 0.01^ab^	1.19 ± 0.11^ab^	0.47 ± 0.02^b^	2.51 ± 0.13^a^	6.16 ± 0.04
SA	6.7 ± 3.9^c^	High	43.07 ± 2.27^de^	2.01 ± 0.05^bc^	0.21 ± 0.01^b^	1.05 ± 0.09^b^	1.53 ± 0.08^a^	0.63 ± 0.03^a^	2.42 ± 0.08^ab^	6.33 ± 0.02
PA	6.7 ± 6.7^c^	High	51.71 ± 4.92^de^	1.93 ± 0.05^bc^	0.27 ± 0.01^ab^	1.08 ± 0.05^b^	1.30 ± 0.02^a^	0.62 ± 0.01^a^	2.09 ± 0.07^ab^	6.39 ± 0.03
OA	57.1 ± 25.7^ab^	Intermediate	23.70 ± 4.92^e^	2.06 ± 0.01^ab^	0.31 ± 0.01^a^	1.45 ± 0.004^a^	1.16 ± 0.27^ab^	0.50 ± 0.07^ab^	2.31 ± 0.24^ab^	6.29 ± 0.06
BG1	60.0 ± 13.3^b^	Intermediate	94.65 ± 7.71^abc^	1.73 ± 0.02^c^	0.31 ± 0.01^a^	1.19 ± 0.1^ab^	1.27 ± 0.06^ab^	0.47 ± 0.03^b^	2.69 ± 0.03^a^	6.01 ± 0.09
FEL	60.0 ± 20.4^ab^	Intermediate	79.06 ± 2.21^bcd^	2.06 ± 0.05^ab^	0.31 ± 0.02^a^	1.19 ± 0.05^ab^	1.35 ± 0.07^a^	0.54 ± 0.02^ab^	2.51 ± 0.20^a^	6.41 ± 0.09
BG4	75.6 ± 4.4^a^	Low	109.64 ± 9.51^ab^	1.92 ± 0.09^bc^	0.27 ± 0.02^ab^	1.17 ± 0.04^ab^	1.18 ± 0.15^ab^	0.48 ± 0.02^b^	2.44 ± 0.20^ab^	6.13 ± 0.02
BA	93.3 ± 6.7^a^	Low	40.67 ± 8.65^e^	2.32 ± 0.06^a^	0.25 ± 0.03^ab^	1.16 ± 0.02^ab^	0.77 ± 0.04^b^	0.43 ± 0.03^b^	1.82 ± 0.09^b^	6.13 ± 0.11
P-Value	< 0.0001		< 0.0001	< 0.0001	0.0023	0.008	0.009	< 0.0001	0.0085	0.05
F Ratio	9		18.33	8.09	4.64	3.71	3.57	6.1	3.64	2.41

Values are means of three replicates ± standard error.

Different letters within the same column indicate significant differences at *p* < 0.005.

**Table 3:** Aboveground traits (biomass and leaf nutrient contents), rhizosphere microbial diversity (Shannon Index) and mortality of strawberry cultivars inoculated with *Macrophomina phaseolina*.

	Resistance	Mortality (%)	Plant biomass (g)	Leaf N (%)	Leaf P (%)	Leaf K (%)	Leaf Ca (%)	Leaf Mg (%)	Leaf Ca:Mg	Shannon Index
GNA	High	10.7 ± 4.6^e^	62.13 ± 5.02^c^	2.02 ± 0.06^ab^	0.23 ± 0.01	1.12 ± 0.04^abc^	1.15 ± 0.1^bcd^	0.50 ± 0.02^bc^	2.30 ± 0.14^bc^	6.29 ± 0.08
MS	High	11.3 ± 3.1^e^	101.12 ± 11.15^ab^	1.98 ± 0.10^ab^	0.26 ± 0.02	1.24 ± 0.07^a^	1.09 ± 0.06^d^	0.45 ± 0.01^c^	2.43 ± 0.11^abc^	6.33 ± 0.04
PA	Intermediate	25.14 ± 7.3^de^	73.03 ± 4.67^bc^	2.08 ± 0.09^ab^	0.26 ± 0.03	1.06 ± 0.11^abc^	1.38 ± 0.08^abcd^	0.62 ± 0.02^ab^	2.24 ± 0.12^c^	6.16 ± 0.11
BG4	Intermediate	30.6 ± 5.2^cde^	101.66 ± 3.00^ab^	1.99 ± 0.10^ab^	0.21 ± 0.01	0.96 ± 0.04^abc^	1.24 ± 0.05^bcd^	0.51 ± 0.02^bc^	2.41 ± 0.07^abc^	6.18 ± 0.07
DR	Intermediate	36.3 ± 2.5^cde^	53.57 ± 2.50^c^	2.14 ± 0.07^ab^	0.30 ± 0.02	1.11 ± 0.01^abc^	1.55 ± 0.03^abc^	0.57 ± 0.01^abc^	2.71 ± 0.08^abc^	6.33 ± 0.10
ABN	Intermediate	55.0 ± 4.4^abc^	57.42 ± 6.14^c^	2.06 ± 0.07^ab^	0.26 ± 0.01	0.90 ± 0.04^bc^	1.57 ± 0.05^ab^	0.57 ± 0.03^abc^	2.75 ± 0.06^ab^	6.26 ± 0.12
ELD	Intermediate	68.8 ± 2.6 ^bcd^	120.85 ± 12.22^a^	1.77 ± 0.07^b^	0.28 ± 0.03	1.26 ± 0.03^a^	1.37 ± 0.11^abcd^	0.48 ± 0.02^c^	2.81 ± 0.15^a^	6.23 ± 0.06
MOY	Intermediate	69.1 ± 8.9^bcd^	59.40 ± 6.19^c^	1.89 ± 0.01^ab^	0.26 ± 0.02	0.83 ± 0.05^c^	1.76 ± 0.08^a^	0.65 ± 0.02^a^	2.71 ± 0.06^abc^	6.15 ± 0.13
FEL	Low	81.6 ± 7.4^ab^	69.49 ± 6.87^bc^	2.14 ± 0.04^ab^	0.25 ± 0.01	1.16 ± 0.03^ab^	1.13 ± 0.08^ cd^	0.48 ± 0.03^c^	2.37 ± 0.1^abc^	6.22 ± 0.07
UCJ	Low	92.86 ± 5.1^a^	79.19 ± 12.47^bc^	2.20 ± 0.15^a^	0.26 ± 0.03	1.00 ± 0.15^abc^	1.43 ± 0.19^abcd^	0.55 ± 0.06^abc^	2.58 ± 0.11^abc^	6.13 ± 0.09
P-Value		< 0.0001	< 0.0001	0.036	0.14	0.0013	0.00016	< 0.0001	0.0027	0.8
F Ratio		25.65	8.4	2.38	1.67	4.26	5.59	6.16	3.81	0.58

Analysis of variance was used to compare groups.

Different letters within the same column indicate significant differences at *p* < 0.005.

Four of the 16 cultivars were represented in both pathogen trials to test the stability of rhizosphere plant-microbial interactions and whether the selection of microorganisms would lead to similar rhizosphere microbial communities, regardless of the environmental conditions and bulk soil microbial community. Significant differences in the microbiome were observed among cultivars (Pseudo-F = 1.83, *p* = 0.001, Fig. S1), and pathogen trial (Pseudo-F = 9.6, *p* = 0.001, Fig. S1), but no significant interaction was found between cultivar and pathogen trial (Pseudo-F = 1.11, *p* = 0.257), indicating the selection of microbes by cultivar in the rhizosphere depended strongly on the environmental conditions and the microbiome of the surrounding bulk soil.

### Relationship between the rhizosphere microbiome and health of the strawberry cultivars

**Figure 5:**
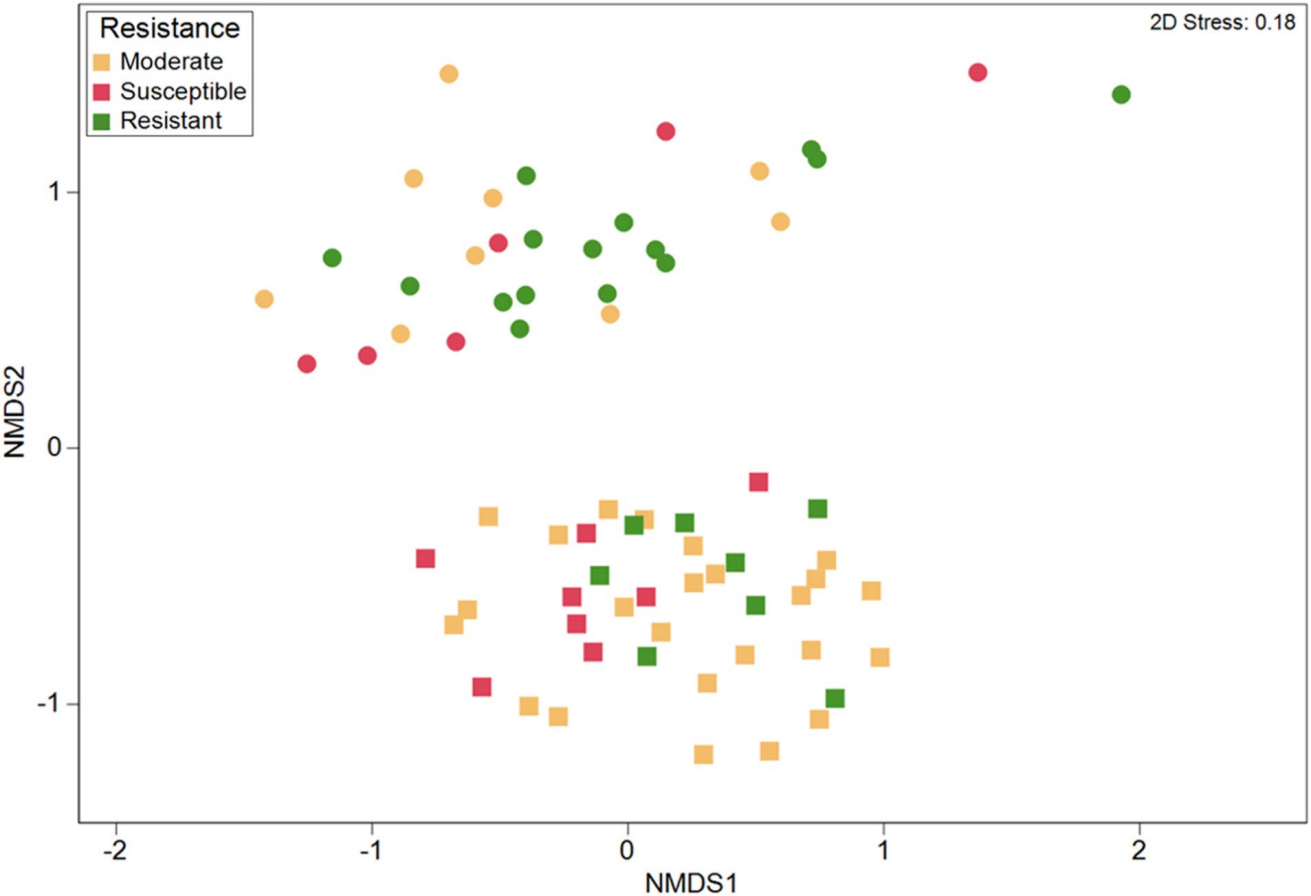
Non-metric multidimensional scaling (NMDS) ordinations based on the Bray–Curtis similarity of bacterial and archaeal OTU community structure among strawberry cultivars exhibiting different resistance against *Verticillium dahliae* (squares) and *Macrophomina phaseolina* (circles).

**Figure 6:**
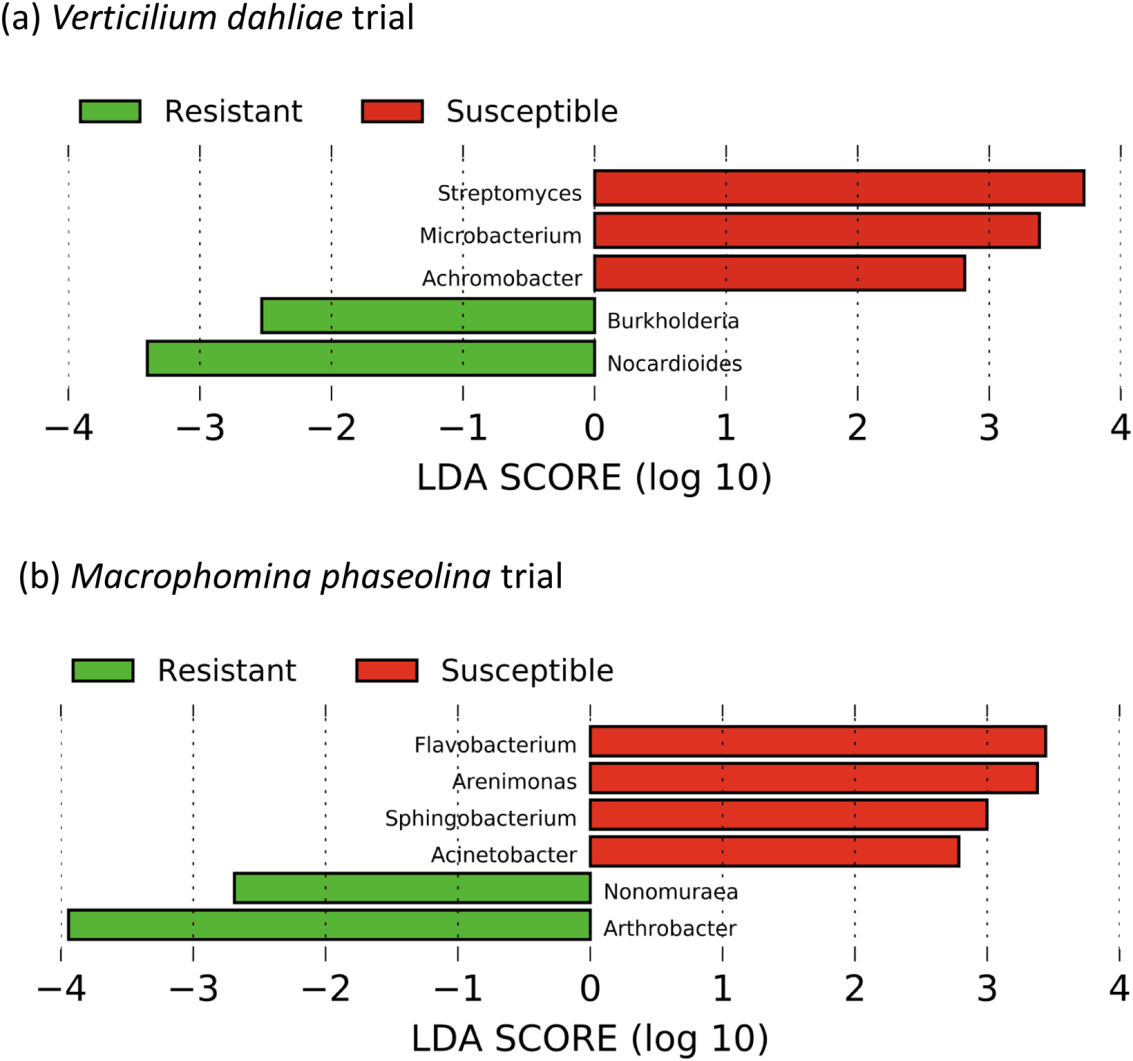
Linear discriminant analysis (LDA) effect size (LEfSe) showing the differential abundance of genera known to include species antagonistic to filamentous fungal pathogen in the rhizosphere of strawberry plants with high and low resistance against *Verticillium dahliae* (**a**) and *Macrophomina phaseolina* (**b**). Genera listed were significantly different (α = 0.05) and had an LEfSe score of > 2.0. A negative LDA score represents depletion in the rhizosphere of low resistance cultivars and enrichment in the rhizosphere of resistant cultivars (green) and a positive LDA score represents the opposite (red).

The ten cultivars selected within each pathogen trial, showed remarkable differences in their mortality or resistance to the soil-borne fungal pathogens, aboveground biomass and nutrient uptake. Three distinct disease resistance groups were observed in the *V. dahliae* trial, with five cultivars showing mortality lower than 10% (resistant), three cultivars showing resistance between 50–70% (moderate) and two cultivars showing more than 70% mortality (susceptible) (Table 2). These differences in plant resistance were matched with differences in the rhizosphere microbiome between the three groups (ANOSIM, R = 0.189, *p* = 0.015, Fig. 5). We further explored the relationship among the rhizosphere microbiome and disease resistance by comparing the microbiome between the two susceptible and the five resistant cultivars. A SIMPER analysis conducted at the genus level, showed that the resistant and susceptible cultivars in the *V. dahliae* field trial differed in 31% of the OTUs, and 23 genera contributing to at least 1% of the differences (Table 4). Twelve genera were more abundant in the resistant cultivars including *Gaiella*, unclassified Acidobacteria *Gp4*, *Gp6*, *Gp16*, *Novosphingobium*, *Arthrobacter*, *Sphingomonas*, *Nocardioides*, *Rubrobacter*, *Variovorax*, *Phycioccus* and *Pseudonocardia* (Table 4). The archaeal genera *Nitrososphaera*, *Streptomyces*, *Rhizobium*, *Pseudomonas* or *Flavobacterium* were more abundant in the susceptible cultivars (Table 4). The LEfSe analysis showed a total of 10 clades that were significantly more abundant in the resistant cultivars as compared to the susceptible (Table S3), including *Burkholderia* and *Nocardioides*, two known fungal antagonists (Fig. 6a).

**Table 4:** SIMPER analysis showing the average percent relative abundances of bacterial genera contributing to at least 1% to the observed variability in the rhizosphere microbiome between cultivars with high and low resistance to *Verticilium dahliae* and *Macrophomina phaseolina*.

Genus	Resistant	Susceptible	Diss/SD	Contribution (%)
***Verticilium dahliae field trial***				
*Nitrososphaera*	7.62	8.24	1.1	9.85
*Gp6*	7.24	6.13	1.1	5.59
*Gaiella*	3.87	3.37	1.25	3.66
*Novosphingobium*	4.66	4.33	1.09	2.99
*Gp16*	2.59	2.37	1.55	2.54
Arthrobacter*	3.3	2.46	1.28	2.38
Streptomyces*	2.31	3.46	1.38	2.35
Rhizobium*	2.4	3.08	1.36	2.28
Pseudomonas*	1.05	1.91	1.31	2.23
Sphingomonas*	4.48	4.3	1.24	2.18
*Gp4*	2.53	1.63	1.29	2.15
*Mucilaginibacter*	2.07	2.58	1.33	2.13
*Phenylobacterium*	2.82	2.88	1.41	1.71
*Hydrocarboniphaga*	0.8	0.96	1.24	1.67
*Steroidobacter*	1.49	1.73	1.42	1.56
*Aminobacter*	1.09	1.27	1.09	1.43
Nocardioides*	2.44	1.86	1.48	1.29
Flavobacterium*	0.81	1.35	0.73	1.26
*Rubrobacter*	1.1	0.55	0.69	1.21
*Variovorax*	1.48	1.38	1.26	1.14
*Phycicoccus*	1.21	1.16	1.3	1.05
*Neorhizobium*	1.21	1.64	1.45	1.04
*Pseudonocardia*	2.21	1.96	1.37	1.04
***Macrophomina phaseolina field trial***				
Pseudomonas*	8.12	7.65	0.91	8.77
*Gp6*	8.05	5.29	1.33	6.02
Arthrobacter*	5.27	3.67	1.4	4.26
Sphingomonas*	4.11	4.42	1.23	3.09
*Novosphingobium*	4.15	4	1.24	2.8
*Gp16*	2.95	2.51	1.32	2.7
*Phenylobacterium*	2.88	3.11	1.36	2.19
*Steroidobacter*	1.63	2.06	1.32	2.11
*Xanthomonas*	0.41	1.34	1.18	1.91
*Nitrososphaera*	2.65	1.82	1.63	1.8
*Gp4*	2.04	1.28	1.34	1.78
Flavobacterium*	0.53	1.23	0.96	1.41
Nocardioides*	2.13	2.1	1.35	1.32
*Mucilaginibacter*	1.38	1.74	1.43	1.28
*Aminobacter*	0.76	1.34	1.09	1.23
Streptomyces*	2.32	2.08	1.04	1.2
*Microvirga*	2.05	1.83	1.2	1.2
Rhizobium*	2.3	2.55	1.45	1.12
*Gaiella*	1.73	1.41	1.57	1.07
*Isoptericola*	0.42	0.61	1.11	1.04

Diss/SD values represent the average dissimilarity divided by the standard deviation.

*Potential fungal antagonists^19^.

**Figure 7:**
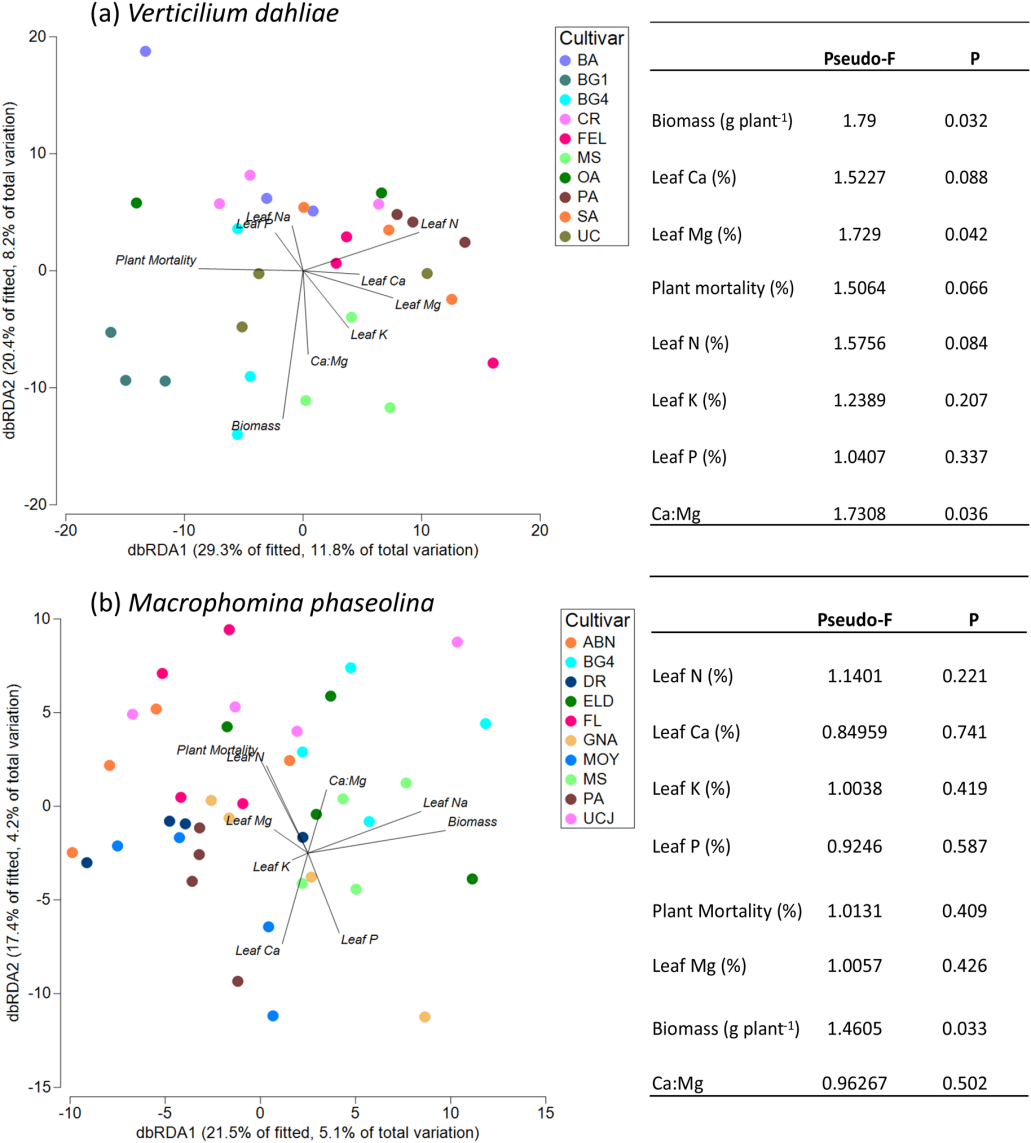
Distance-based redundancy analysis (dbRDA) on rhizosphere bacterial and archaeal communities at OTU level and selected plant traits in the *Verticillium dahliae* (**a**) and *Macrophomina phaseolina* (**b**) trials.

No significant correlation was found between the diversity of the rhizosphere microbiome and plant mortality in response to *V. dahliae* (*p* = 0.06), although the Shannon diversity index was significantly positively correlated to the contents of Ca (*p* = 0.04) and Mg in the leaves of the strawberry plants (*p* = 0.004; Table S5). We further explored the relationship between the rhizosphere bacterial and archaeal community, plant mortality and other aboveground plant traits by using distance-based redundancy analysis (db-RDA). We observed that microbial community composition in the rhizosphere of the strawberry plants grown in the presence of *V. dahliae*, was significantly associated with aboveground plant biomass, the leaf Mg content as well as the leaf Ca:Mg ratio of the plants (Fig. 7a).

Differences in plant mortality among the cultivars in the *M. phaseolina* trial were significant but not as clear as in the *V. dahliae* trial, with a susceptible (more than 80% mortality), a moderate (20–70% mortality), and a resistant group (< 20% mortality) (Table 3). No differences among these resistance groups were found in their rhizosphere microbiome (R = − 0.048, *p* > 0.05, Fig. 5). We further explored the relationship among the rhizosphere microbiome and disease resistance by comparing the microbiome between the two cultivars with the highest mortality rates (FL, UCJ) and the two cultivars with the lowest mortality (GNA, MS). We found significant differences in the overall OTU community between susceptible and high resistant cultivars (R = 0.2, *p* = 0.009). A SIMPER analysis on these cultivars (conducted with taxa grouped at the genus level) found an average dissimilarity of 28.8% between susceptible and resistant cultivars. A total of 20 genera contributed to at least 1% of the differences between resistant and susceptible cultivars (Table 4). Eleven genera were more abundant in the resistant cultivars, including *Pseudomonas*, *Gp6* and *Arthobacter*, which contributed to the largest differences, as well as *Novosphingobium*, *Gp16*, *Nitrososphaera*, *Gp4*, *Nocardioides*, *Streptomyces*, *Microvirga* and *Gaiella*. On the other hand, the genera *Sphingomonas*, *Phenylobacterium*, *Xanthomonas*, *Flavobacterium*, *Mucilaginibacter*, *Aminobacter*, *Rhizobium* and *Isoptericola* were all more abundant in the rhizosphere of the cultivars susceptible to *M. phaseolina* (Table 4)*.* LEfSe analysis revealed that resistant cultivars had 34 clades that were significantly more abundant as compared to susceptible cultivars (Table S4), including the fungal antagonists *Arthobacter* and *Nonomuraena* (Fig. 6b). The fungal antagonistic bacteria from the genus *Flavobacterium*, *Arenimonas*, *Sphingobacterium* and *Acinetobacter* were more abundant in the rhizosphere of susceptible plants.

Plant mortality was not found to be significantly correlated to plant aboveground biomass, leaf nutrient content or to the microbial diversity in the rhizosphere of the *M. phaseolina* trial (Table S6). Plant biomass was significantly positively correlated to leaf K content (*p* < 0.001), and some other significant correlations were found between leaf K and leaf Ca, Mg and P, although none of these leaf nutrients were correlated to the rhizosphere microbial diversity (Shannon index). The dbRDA analysis revealed that plant biomass was significantly associated with the composition of the microbiome in the rhizosphere, but not with the plant leaf nutrient contents (Fig. 7b).

## Discussion

Understanding plant-microbial interactions is instrumental for the development of low input management strategies in high value crops. Here we studied the role of the rhizosphere bacterial and archaeal community in the growth and health of strawberry plants subjected to the biotic stress imposed by two different soil-borne fungal pathogens. To do this, we used 16 cultivars with remarkably different phenotypes including aboveground biomass, nutrient uptake and resistance to soil-borne diseases and we grew them under commercial field conditions. As we had initially hypothesized, the rhizosphere microbiome of the strawberry plants was clearly different from the soil microbiome in the two pathogen trials, showing that the plants are able to select from the pool of microorganisms available in the soil. This has been observed in many other plant species such as rice, corn, tomatoes, beans, barley and *Arabidopsis*^34,37–42^; to the best of our knowledge, the research presented here constitutes the first evidence of a distinct rhizosphere microbiome in different strawberry cultivars and environmental conditions. The differences between bulk and rhizosphere soil were observed consistently in the two pathogen trials which had a different soil management history and a different soil microbiome. Interestingly, the structure and diversity of the rhizosphere microbiome of the strawberry cultivars in the two soils were more similar to each other than to their respective bulk soils. This suggests that plant roots, even though they can only select from the pool of microbes available in their surrounding environment, exhibit strong and consistent selection regardless of environmental conditions. This rhizosphere filter has been attributed to the change in pH, redox conditions and production of rhizodeposits including the release of root exudates of different nature^7,8,43,44^.

The relative abundance of most bacterial and archaeal phyla was lower in the rhizosphere than in the surrounding bulk soil with the exception of Proteobacteria, as has been previously observed in other plant species^42,45^, as well as Verrucomicrobia and Bacteroidetes in the *V. dahliae* trial. The phyla Proteobacteria and Bacteroidetes include several bacteria that are known to promote plant growth and/or to be antagonists of soil-borne fungal pathogens, such as *Rhizobium, Pseudomonas, Burkholderia* and *Flavobacterium*^19,46^. All of these taxa, along with other known fungal antagonists, were found in higher abundances in the rhizosphere of the strawberry plants. This capacity of the plants to recruit beneficial microorganisms that help them cope with biotic and abiotic stress has been reported previously for other crops^5,47,48^. The genus *Pseudomonas* is known to include several plant growth promoting species and strains capable of producing plant hormones such as auxins, induce systemic resistance, and produce cell wall degrading enzymes and siderophores that chelate iron^18,19,49^. Similarly, rhizosphere free-living species from the genus *Rhizobium* have been reported to produce antibiotic substances, chelates and induce systemic resistance in a wide range of crops^50,51^. Interestingly, the rhizosphere of the strawberry plants was enriched with bacteria that are known to be able to fix atmospheric N, such as *Rhizobium* and *Burkholderia. Rhizobium* includes N-fixing bacteria known to establish symbiotic interactions in legumes, however recent evidence shows that *Rhizobium* could also be naturally present in the rhizosphere of non-legume crops such as sunflower, corn, barley or rice, and can be successfully inoculated in the rhizosphere of vegetables such as tomato, pepper, lettuce and radish, increasing their growth significantly^52,53^. The genus *Burkholderia* includes several diazotrophic species, which have been recently found to play a significant role in nitrogen fixation in tomatoes, corn and sugarcane^54–56^.

In addition to the differences in the overall structure of the microbiome, microbial diversity was different in the rhizosphere of the strawberry plants compared to the bulk soil. Consistent with previous studies that show a decrease in microbial diversity closer to the roots^39,45^, a lower microbial diversity was observed in the rhizosphere of the plants grown in the *M. phaseolina* trial. The opposite trend was observed in the *V. dahliae* trial. Despite this, no significant differences were found in rhizosphere-associated microbial diversity between the two pathogen trials, again showing the homogenizing effect of the microbial filter of the rhizosphere. Similar results were observed by Mendes et al.^34^ when growing common bean in two different soils.

We assessed whether the presence of the soil-borne fungal pathogens increased the abundance of beneficial bacteria in the rhizosphere of the strawberry plants. The presence of *V. dahliae* has been shown to induce a change in the fungal microbial community of strawberry in a previous field study^57^. In our study, the rhizosphere microbiome was significantly modified in the treatment versus the control plots with increases in the relative abundance of the actinobacteria *Pseudonocardia* and *Gaiella* as well as the ammonia-oxidizing archaea *Nitrososphaera* in the plots with *V. dahliae*; and increases in the relative abundance of *Povalibacter,* a saprophytic proteobacterium, and *Pseudomonas* in the plots inoculated with *M. phaseolina*. These changes could be attributed to a change in the root exudation patterns in the presence of the soil-borne pathogens, a higher prevalence of dead roots, and microbial competition^7,10,58,59^. Nevertheless, it is also possible that the different management (i.e. fumigation) in the case *V. dahliae* controls versus the inoculated plots, contributed to the observed differences in this field.

Differences in rhizosphere microbiome between the cultivars showed genotype-dependent selection of the rhizosphere microorganisms as has been observed in other plant species^39,41^. However, comparison of the same cultivars grown parallelly in the two pathogen trials, showed that their microbial communities changed depending on the field where they were grown, suggesting strong genotype by environment interactions. In fact, these same cultivars showed very different leaf nutrient concentrations and mortality rates, that could be driven by the different soil-borne pathogens, initial soil fertility and microbiome in both pathogen trials^60^.

Previous studies have shown that, cultivar-specific rhizosphere microbiomes are associated with increased resistance to soil-borne diseases. Resistance can be due to the presence of biocontrol taxa that directly or indirectly suppress the disease (specific disease suppression), and plant growth promoting bacteria that induce systemic disease resistance in the plant. However it is also possible that disease suppression is the result of the coordinated action of all the microorganisms present in the rhizosphere (general disease suppression)^61^. Mendes et al.^34^ studied the rhizosphere microbiome of different common bean cultivars with different degrees of resistance to *Fusarium oxysporum*, and observed that a resistant cultivar had a higher abundance of certain bacterial families such as Pseudomonadaceae, Bacillaceae, Solibacteraceae and Cytophagaceae. Using a combination of 16S rRNA sequencing, shotgun metagenomics, and plate cultures, Kwak et al.^36^ were able to determine that the resistance of the tomato cultivar Hawaii 7996 to the soil-borne pathogen *Ralstonia solanacearum* was conferred by the presence of a *Flavobacterium* strain. In our study, comparisons in the rhizosphere microbiome between cultivars with different levels of resistance against the two fungal pathogens showed significant differences. Correlation analysis carried out through db-RDA showed no relationship between the whole community of the rhizosphere and plant mortality suggesting that resistance could be associated to the presence of biocontrol taxa but not general suppression of the soil-borne diseases. Taxa found in higher abundance in the cultivars resistant to *V. dahliae* included, *Burkholderia*, *Sphingomonas and Novosphingonium* which have been found to suppress this fungal pathogen in previous studies^19,62^*.* Additionally, higher abundances of unclassified Acidobacteria (Gp6, Gp16 and Gp4) and Actinobacteria (*Arthrobacter, Nocardioides* and *Gaiella*) were found in resistant strawberry cultivars. Acidobacteria have been found to correlate well with apple tree growth before^63^ and to be negatively correlated to the abundance of *Fusarium oxysporum* in banana^64^. *Arthrobacter* is a known antagonist to *V. dahliae* through the production of cell wall degrading enzymes^19,65^, whereas *Nocardioides* and *Gaiella* have recently shown to be essential to the resistance of strawberry plants against *F. oxysporum*^66^. A recent study performed with soils collected at the continental scale, found that acidobacteria and actinobacteria are key players in the suppression of the fungal pathogen *Fusarium oxysporum*^67^. Results of this study show that both acidobacteria and actinobacteria are also key taxa in the resistance of strawberry cultivars against *V. dahliae*.

In the *M. phaseolina* trial, resistant cultivars were characterized by greater abundances of *Pseudomonas*, actinobacteria (*Nonomuraea*, *Arthrobacter, Streptomyces, Nocardioides* and *Gaiella*), which are well-known to be fungal antagonists and plant growth promoters^19,48,68^. Additionally, the rhizosphere of resistant plants was also characterized by higher abundances of unclassified Acidobacteria (Gp6, Gp16 and Gp4). Interestingly, the same taxa seemed to be in higher abundances in the resistant cultivars in the two field trials, showing a clear role of the rhizosphere microbiome and suggesting a common mechanism in the resistance to soil-borne diseases in the strawberry cultivars.

In addition to playing a role in plant resistance to biotic stress, the rhizosphere microbiome was significantly correlated to other plant traits including plant biomass and leaf Mg and Ca:Mg ratio in the *V. dahliae* field trial suggesting a role in the response to abiotic stress. The soil at the experimental fields had a high concentration of Mg and low Ca: Mg ratio that could create nutrient stress in the plants as shown by the significant correlations between leaf Mg, Ca, Ca:Mg with plant mortality. Together, these findings show a role of the rhizosphere microbiome in regulating the response of the plant to biotic and abiotic (nutritional) stress. The specific mechanisms and plasticity of these beneficial plant-microbial interactions needs to be further investigated. In particular, given the strong genotype by environment interactions observed when cultivars were compared across the two fields, it is necessary to determine what are the optimal soil conditions and management strategies that can promote beneficial plant-microbial interactions.

## Conclusions

Strawberry plants can recruit microorganisms to their rhizosphere from the pool of microbes available in the soil, increasing the relative abundance of beneficial microorganisms involved in nutrient uptake and resistance to soil-borne diseases. Results of two field trials using 16 cultivars show a higher abundance of diazotrophic bacteria in the rhizosphere of strawberry plants which should be further explored as a strategy to reduce inputs of N fertilizers. Furthermore, cultivars resistant to the two soil-borne pathogens consistently showed higher abundances of biocontrol microorganisms such as *Pseudomonas*, and actinobacteria such as *Arthrobacter, Nocardioides* and *Gaiella.* Selection of rhizosphere microorganisms is strongly associated to the different genotypes and therefore seems to be a genetic trait that could be selected through breeding; nevertheless, the microbiome selected by a specific cultivar depends largely on the environmental conditions and microbiome of the surrounding soil. Thus, this suggests that soil health may be important in the establishment of these beneficial plant-microbial interactions.

## Material and methods

### Plant material

Strawberry cultivars from different breeding programs and representing a range of resistance to soil-borne fungal diseases, were selected from two large ongoing field trials, evaluating 90 cultivars and elite selections for their resistance to *Macrophomina phaseolina* and *Verticillium dahliae.* These trials were established in October through July in 2015–2016, 2016–2017 and 2017–2018. Plant material for the current study was selected from the two pathogen trials established in October 2016. We selected 10 cultivars exhibiting a range of resistance, from high to low, to *Macrophomina phaseolina* and *Verticillium dahliae*. Out of the 10 cultivars, 6 were unique to each trial and 4 were grown in both trials, for a total of 16 different cultivars evaluate in this study. The names and breeding programs of the cultivars selected from each field trial are shown in Table S7.

### Field trials

Two parallel (located 0.4 km apart) replicated pathogen trials were established on the California Polytechnic State University campus in San Luis Obispo, California (USA) to evaluate the 90 strawberry cultivars and elite selections for their resistance to the soil-borne fungal pathogens. The soil at both field sites was classified as Pachic Haploxeroll on a 0–2% slope with a clay loam texture. The soil in the *V. dahliae* trial had a pH of 7, electrical conductivity (EC) of 1.2, cation exchange capacity (CEC) of 25.5, and 3% organic matter. The soil in the *M. phaseolina* trial had a pH of 6.8, EC of 1.6, CEC of 17.4, and 3% organic matter.

The trials followed a complete randomized block design with four blocks. Briefly, in each trial, strawberry plants were grown in 1.6 × 2.5 m plots established in raised beds (0.3 m high). Plots contained 20 plants of the same cultivar and constituted the experimental unit. Ninety plots (one per cultivar) were arranged randomly within each block and 4 replicates (blocks) were established (Fig. S1). This design resulted in a total of 360 plots per field trial (90 cultivars × 4 replicates or beds).

Differences in pathogen history between the two field sites prompted distinct methods for ensuring host infection. The *V. dahliae* trial relied on high levels of microsclerotia found naturally in the field soil (20 CFU g^−1^ soil). The Andersen sampler technique^69^ was used to estimate *V. dahliae* populations in field soil with the following methods. Eight soil cores (2.5 by 30 cm) were collected from each replicated plot, bulked, and thoroughly mixed for analysis. Soil aggregates from each sample were broken up and thoroughly mixed for 5 min. After air drying the soil at room temperature for 1 week, each sample was ground into a fine powder with a mortar and pestle. An Andersen air sampler was used to distribute the soil on the surface of NP-10 medium^70^. Three replicates of 5 total plates were processed for each sample. After 10 days of incubation in the dark at 20 °C, the surface of the agar medium was gently washed under a stream of water to remove soil particles, and microsclerotial colonies of *V. dahliae* were counted with the aid of a dissecting microscope. The estimate of soil inoculum density was expressed as an average number of CFU of *V. dahliae* g^−1^ of dry soil.

*Macrophomina phaseolina* was not naturally present in the soil at the field site and had to be inoculated. Prior to the inoculation, the field was fumigated (392 kg ha^-1^ of a 50% chloropicrin + 50% methyl bromide solution) to ensure the removal of all other soil-borne fungal pathogens. Fumigation was carried out in 2015, two years before the start of this trial, since the effects last usually 2–3 years. At transplanting, 5 g of *M. phaseolina* inoculum (20,500–25,200 CFU g^-1^) was applied to the soil-crown interface. *Macrophomina* inoculum was produced from field isolates collected from diseased plants in 2014 and 2015 following procedure by Mihail^71^. Briefly, a homogenized 1:0.4:0.4 sand: cornmeal: deionized water mixture was autoclaved for one hour on two separate days in separate 250 mL containers (Nalgene, Rochester, NY). After cooling to room temperature, the containers were inoculated with plugs of the pathogen. Inoculated cornmeal-sand containers were then incubated at 30 °C for two weeks and shaken vigorously by hand daily to aid rapid colonization. After incubation the colonized cornmeal-sand inoculum was spread over a metal tray to air dry for five days. Once dry, this inoculum was stored in the dark at room temperature for two weeks before being applied in the field. The density of viable microsclerotia within the cornmeal-sand inoculum was enumerated with direct plating of 10^–3^ to 10^–4^ serial dilutions onto NP-10 medium^70^. Ten replicate plates of each dilution were used. The plates were incubated in the dark at 30 °C for 7 days before counting colony forming units (CFU).

Additionally, in each pathogen trial, one block was established as a non-inoculated control where the soil-borne pathogens were eliminated through chemical fumigation prior to planting (*V. dahliae* trial) or not introduced artificially (*M. phaseolina* trial). The control block contained one 20-plant plot per cultivar for a total of 90 plots (90 cultivars). Due to the lack of replication, no comparisons were made between inoculated and control plots at the cultivar level. Nevertheless, control plots were used to assess the overall effects of pathogen presence in the rhizosphere microbiome of the plants.

Bare-root strawberry seedlings were transplanted to the control and experimental plots in mid-October 2016 and grown until the end of July 2017. Fertilization was carried out through subsurface drip irrigation (fertigation) during the growing season and preplant slow release fertilizer banded in the bed for a total input of 112 kg N ha^−1^.

### Soil and plant sample collection

During fruit set, we determined plant mortality in the 90 cultivars visually by assessing the percentage of dead plants in each plot. Additionally, presence of the pathogens in symptomatic plants was confirmed by plating tissue pieces of internal crown tissue on selective media. Tissue isolations were conducted on 10 symptomatic plants in each replication in the host resistance trial on a biweekly basis. Subsequently, we selected ten representative cultivars exhibiting a range of high to low resistance within each pathogen trial for further study of their rhizosphere microbiome. A summary of the cultivars used in this study with their respective breeding programs is shown in Table S7.

At harvest (July 2017), we performed another visual evaluation to determine actual plant mortality per plot at the moment of harvest (% of dead plants). Plants were classified as susceptible (more than 70% mortality), moderate (20–70% mortality), and resistant (< 20% mortality). One shoot, soil and rhizosphere composite sample was collected subsequently from each plot for each of the selected cultivars (Fig. S2). Briefly, within each plot, four plants were randomly selected while avoiding dead or dying plants to reduce the confounding effects of the dead root tissue, typical of symptomatic plants, in the soil microbiome. The above and belowground components of each plant were separated and then consolidated separately by plot to obtain one root and one shoots sample per plot. Fresh shoot biomass was recorded in the field after removing fruit. Subsamples of the shoot biomass were then collected in each plot for oven drying and the determination of aboveground plant biomass and leaf nutrient content. A preliminary sampling showed that the highest concentration of primary and secondary roots was found in a radius of 5 cm from the crown and 10 cm from the soil surface. Root samples were collected using 5-cm diameter cores centered around the strawberry crown to a depth of approximately 10 cm. A 750 cm^3^ bulk soil sample (without the presence of roots) from each plot was also collected from the center of each strawberry bed (50 cm from the nearest plant) at a depth of 10 cm. Rhizosphere soil was collected by dry sieving roots on sterilized 500 µm sieves. Samples were lyophilized and stored at − 80 °C prior to DNA extraction.

### Leaf tissue analysis

Tissue analysis was performed using leaf and petiole samples dried for 24 h at 55 °C. Dry samples were crushed using a mortar and pestle, followed by two 2-min cycles of grinding in a Bel-Art Micro-Mill grinder (Wayne, NJ, USA). Tissue carbon and nitrogen concentrations were analyzed from a 300 mg subsample using a vario MAX CNS Macro Elemental Analyzer (Elementar Analysensysteme GmbH, Hanau, Germany). Quality control was ensured by analyzing duplicate samples and NIST SRM 1573A tomato leaf standards every ten samples. Concentrations of leaf P, K, Ca and Mg were determined using a modified USEPA^72^ acid digestion method in the Ultima 2 ICP-OES (Horiba Jobin Yvon S.A.S., Logjumeau, France).

### DNA isolation and extraction from rhizosphere and bulk soils

To study bacterial and archaeal diversity in the soil and rhizosphere samples, DNA was extracted from a 250 mg subsample using the DNeasy PowerSoil Kit (QIAGEN, Venlo, Netherlands) following the manufacturer’s recommendations. DNA yield was measured on a NanoDrop 2000 (Thermo Fisher Scientific, Waltham, MA, USA). Samples with yield below 5 ng/µL were extracted again prior to amplification. The V4 region of the 16S rRNA was subsequently sequenced using bacterial/archaeal universal primers 515F (5′ -GTG YCA GCM GCC GCG GTA A- 3′) and 806R (3′ -GGA CTA CNV GGG TWT CTA AT-3′)^73^. PCR reagents included 25 µL of DDH20, 2.5 µL of each primer, 12.5 µL Phusion Hot start flex 2 × Master Mix (Thermo Fisher Scientific), and 50 ng of template DNA. The thermal cycling scheme consisted of 30 s at 98 °C, 10 s at 98 °C, 35 cycles of 54 °C for 30 s, 45 s at 72 °C and 10 min at 72 °C. After the first round of PCR, sequencing adapters and barcodes were added for further amplification. PCR amplification products were visualized by 2% agarose gel electrophoresis. The target fragments were recovered using the AxyPrep PCR Cleanup Kit (Axygen Biosciences, Union City, CA, USA) according to manufacturer’s instructions. The PCR product was further purified using the Quant-iT PicoGreen dsDNA Assay Kit (Thermo Fisher Scientific). The library was quantified on the QuantiFluor fluorescence quantification system (Promega Corporation, Madison, WI, USA). Each qualified sequencing library (i.e., had concentrations above 2 mM) was diluted and pooled/multiplexed. The pooled library was loaded on Illumina MiSeq and the MiSeq Reagent Kit v2, 500 cycles (Illumina) was used for paired-end sequencing (2 × 250 bp). Raw sequence data has been deposited in GenBank (accession number PRJNA666409).

### Sequence data processing

Paired-end sequences obtained on the MiSeq platform were de-multiplexed, followed by raw data filtering and processing utilizing the UPARSE pipeline (v11.0.667, www.drive5.com/usearch). Paired end reads were merged with an allowance of 10 bp max differences and with a maximum allowance of 10 bp differences and > 80% identification. Sequences with less than 50 bp were filtered and all sequences trimmed to 280 bp. Low quality sequences were filtered using a maximum expected error threshold of 1.0. Sequences were clustered into *denovo* Operational Taxonomic Units (OTUs) based on 97% similarity and singletons were discarded. Taxonomy was assigned to representative OTU sequences using the Naïve Bayesian classifier^74^ based on the Ribosomal Database Project^37,75^. Chloroplast and unassigned sequences were removed from OTU tables prior to statistical analyses. Samples were rarefied to match the sequences of the lowest count sequence of 6,666 for analyses using taxonomic rank abundance.

### Data analysis

Resemblance matrices for the OTU tables were generated using Bray–Curtis similarity of the standardized and Log (x + 1) transformed data^76^. Interpretations of multivariate distances were conducted using nonmetric multi-dimensional scaling (NMDS) ordinations based on the resemblance matrices using a maximum of 50 restarts and the lowest stress solutions. The differences between sample types (rhizosphere or bulk soil), pathogen trials (*V. dahliae* or *M. phaseolina*), plant genotypes and resistance to the soil-borne diseases in the soil microbial community composition within each pathogen trial was assessed through analysis of similarities (ANOSIMS) or PERMANOVA (for bifactorial analyses) using Spearman rank correlation with 999 max permutations of the resemblance matrices were used to determine significance levels between groups. Similarity percentage (SIMPER) analyses were conducted using the Bray–Curtis similarity matrix. Correlations between rhizosphere OTU relative abundances and plant variables (leaf nutrient contents and mortality) were made using the distance-based linear model (DistLM) with 999 permutations. All microbial community data were analyzed using PRIMER-e version 7 with the PERMANOVA + addon (Quest Research Limited, Auckland, NZ).

The effect of ‘sample type’ (bulk vs. rhizosphere soil), ‘cultivar’ and ‘cultivar resistance’ to the fungal pathogens on the microbial diversity (Shannon diversity index), plant mortality, and plant nutrient comparisons among groups were conducted using general linear models and Tukey HSD post hoc tests in JMP Pro (SAS, Cary, NC); Pearson’s correlations were carried out among the different plant traits analyzed using JMP Pro 13. A list of bacterial genera known to include species antagonistic to filamentous fungal plant pathogens was used to compare treatment groups (Inderbitzin et al., 2017). Linear discriminant analysis (LDA) effect size (LEfSe) comparing taxonomic features between groups was conducted online (www.huttenhower.sph.harvard.edu/galaxy) with an LDA threshold of 2.0 and an alpha value of 0.05.

## Supplementary Information

Supplementary Information.
