# Supplementary material for: The rhizosphere microbiome plays a role in the resistance to soil-borne pathogens and nutrient uptake of strawberry cultivars under field conditions
Source: Sci Rep. 2021 Feb 4;11:3188. doi: 10.1038/s41598-021-82768-2 (PMC7862632; doi:10.1038/s41598-021-82768-2)
Supplement: Supplementary file 1 — Supplementary Information. [file 41598_2021_82768_MOESM1_ESM.docx]

**The rhizosphere microbiome plays a role in the resistance to soil-borne pathogens and nutrient uptake of strawberry cultivars under field conditions**

Cristina Lazcano^1*^, Eric Boyd^2,3^, Gerald Holmes^3^, Shashika Hewavitharana^3^, Alexis Pasulka^4^, Kelly Ivors^3^

1. Department of Land, Air and Water Resources, University of California Davis. One Shields Avenue, Davis, CA 95616-8627, USA

2. Department of Natural Resources Management and Environmental Sciences. California Polytechnic State University, San Luis Obispo, CA 93407, USA

3. Strawberry Center California Polytechnic State University, San Luis Obispo, CA 93407, USA

4. Biological Sciences Department, California Polytechnic State University, San Luis Obispo, CA 93407, USA

***Corresponding author:**

[clazcano@ucdavis.edu](mailto:clazcano@ucdavis.edu)

**Supplementary Table 1.** Linear discriminant analysis (LDA) effect size analysis (LEfSe) comparing bacterial/archaeal relative abundance between bulk and rhizosphere soil in the rhizosphere and bulk soils in the *Verticillium dahliae* pathogen trial. Sample type listed was found to have a greater average relative abundance. Parameters were set at α < 0.05, and an effect size of > 2.0.

**Supplementary Table 2.** Linear discriminant analysis (LDA) effect size analysis (LEfSe) comparing bacterial/archaeal relative abundance between bulk and rhizosphere soil in the rhizosphere and bulk soils in the *Macrophomina phaseolina* pathogen trial. Sample type listed was found to have a greater average relative abundance. Parameters were set at α < 0.05, and an effect size of > 2.0

**Supplementary Table 3.** Linear discriminant analysis (LDA) effect size analysis (LEfSe) comparing bacterial and archaeal relative abundance in the rhizosphere of strawberry cultivars with high and low resistance against *Verticilium dahliae*. Parameters were set at α < 0.05, and an effect size of > 2.0.

|  | Resistance | LDA effect size | p-value |
| --- | --- | --- | --- |
| Bacteria.Bacteroidetes.Cytophagia.Cytophagales.Cytophagaceae.Hymenobacter | High | 2.622 | 0.018 |
| Bacteria.Actinobacteria.Actinobacteria.Solirubrobacterales.Solirubrobacteraceae | High | 3.272 | 0.043 |
| Bacteria.Actinobacteria.Actinobacteria.Solirubrobacterales.Solirubrobacteraceae.Solirubrobacter | High | 3.028 | 0.020 |
| Bacteria.Firmicutes.Clostridia.Clostridiales.Peptostreptococcaceae.Sporacetigenium | High | 2.731 | 0.014 |
| Bacteria.Acidobacteria.Acidobacteria_Gp1.Gp1 | High | 2.743 | 0.029 |
| Bacteria.Actinobacteria.Actinobacteria.Actinomycetales.Nocardioidaceae.Nocardioides | High | 3.399 | 0.043 |
| Bacteria.Proteobacteria.Betaproteobacteria.Burkholderiales.Burkholderiaceae.Burkholderia | High | 2.531 | 0.033 |
| Bacteria.Firmicutes.Bacilli.Bacillales.Bacillaceae_2 | High | 2.567 | 0.016 |
| Bacteria.Actinobacteria.Actinobacteria.Actinomycetales.Pseudonocardiaceae.Actinomycetospora | High | 2.739 | 0.010 |
| Bacteria.Proteobacteria.Alphaproteobacteria.Rhizobiales.Rhizobiaceae.Ensifer | High | 2.682 | 0.047 |
| Bacteria.Bacteroidetes | Low | 3.847 | 0.020 |
| Bacteria.Acidobacteria.Acidobacteria_Gp11 | Low | 2.649 | 0.022 |
| Bacteria.Proteobacteria.Gammaproteobacteria.Enterobacteriales | Low | 3.789 | 0.016 |
| Bacteria.Actinobacteria.Actinobacteria.Actinomycetales.Pseudonocardiaceae.Allokutzneria | Low | 2.752 | 0.035 |
| Bacteria.Actinobacteria.Actinobacteria.Actinomycetales.Microbacteriaceae.Microbacterium | Low | 3.380 | 0.013 |
| Bacteria.Proteobacteria.Betaproteobacteria.Hydrogenophilales.Hydrogenophilaceae.Thiobacillus | Low | 2.548 | 0.022 |
| Bacteria.Fusobacteria.Fusobacteriia.Fusobacteriales.Leptotrichiaceae | Low | 2.866 | 0.022 |
| Bacteria.Proteobacteria.Gammaproteobacteria.Enterobacteriales.Enterobacteriaceae | Low | 3.847 | 0.016 |
| Bacteria.Proteobacteria.Alphaproteobacteria.Rhizobiales.Bradyrhizobiaceae.Bosea | Low | 2.756 | 0.036 |
| Bacteria.Proteobacteria.Gammaproteobacteria | Low | 3.943 | 0.020 |
| Bacteria.Proteobacteria.Betaproteobacteria.Hydrogenophilales.Hydrogenophilaceae | Low | 2.540 | 0.022 |
| Bacteria.Fusobacteria.Fusobacteriia | Low | 2.937 | 0.022 |
| Bacteria.Fusobacteria.Fusobacteriia.Fusobacteriales.Leptotrichiaceae.Sebaldella | Low | 2.909 | 0.022 |
| Bacteria.Bacteroidetes.Sphingobacteriia.Sphingobacteriales.Chitinophagaceae.Taibaiella | Low | 2.888 | 0.006 |
| Bacteria.Actinobacteria.Actinobacteria.Actinomycetales.Streptomycetaceae.Streptomyces | Low | 3.720 | 0.029 |
| Bacteria.Fusobacteria.Fusobacteriia.Fusobacteriales | Low | 2.863 | 0.022 |
| Bacteria.BRC1.BRC1_genera_incertae_sedis | Low | 2.878 | 0.022 |
| Bacteria.Proteobacteria.Betaproteobacteria.Burkholderiales.Alcaligenaceae.Achromobacter | Low | 2.813 | 0.031 |
| Bacteria.Bacteroidetes.Sphingobacteriia.Sphingobacteriales.Sphingobacteriaceae.Olivibacter | Low | 2.585 | 0.031 |
| Bacteria.Proteobacteria.Betaproteobacteria.Hydrogenophilales | Low | 2.542 | 0.022 |
| Bacteria.Acidobacteria.Acidobacteria_Gp11.Gp11 | Low | 2.479 | 0.022 |
| Bacteria.Fusobacteria | Low | 3.042 | 0.022 |
| Bacteria.Proteobacteria.Gammaproteobacteria.Enterobacteriales.Enterobacteriaceae.Yersinia | Low | 2.741 | 0.034 |
| Bacteria.Actinobacteria.Actinobacteria.Actinomycetales.Streptomycetaceae | Low | 3.928 | 0.024 |
| Bacteria.Bacteroidetes.Sphingobacteriia | Low | 3.703 | 0.036 |
| Bacteria.Chlamydiae.Chlamydiia.Chlamydiales.Parachlamydiaceae.Parachlamydia | Low | 2.587 | 0.022 |

**Supplementary Table 4.** Linear discriminant analysis (LDA) effect size analysis (LEfSe) comparing bacterial and archaeal relative abundance in the rhizosphere of strawberry cultivars with high and low resistance against *Macrophomina phaseolina*. Parameters were set at α < 0.05, and an effect size of > 2.0.

|  | Resistance | LDA Effect | p-value |
| --- | --- | --- | --- |
| Bacteria.Actinobacteria.Actinobacteria.Actinomycetales.Micrococcaceae | High | 3.984 | 0.046 |
| Bacteria.Actinobacteria.Actinobacteria.Actinomycetales.Micrococcaceae.Arthrobacter | High | 3.963 | 0.046 |
| Bacteria.Acidobacteria.Acidobacteria_Gp4.Gp4 | High | 3.661 | 0.036 |
| Archaea.Thaumarchaeota.Nitrososphaerales.Nitrososphaeraceae | High | 3.603 | 0.021 |
| Archaea.Thaumarchaeota.Nitrososphaerales.Nitrososphaeraceae.Nitrososphaera | High | 3.599 | 0.021 |
| Archaea.Thaumarchaeota.Nitrososphaerales | High | 3.539 | 0.016 |
| Bacteria.Actinobacteria.Actinobacteria.Solirubrobacterales | High | 3.457 | 0.036 |
| Bacteria.Chloroflexi | High | 3.372 | 0.021 |
| Archaea.Thaumarchaeota | High | 3.361 | 0.021 |
| Archaea | High | 3.313 | 0.021 |
| Bacteria.Armatimonadetes.Armatimonadetes_gp2 | High | 3.253 | 0.014 |
| Bacteria.Proteobacteria.Deltaproteobacteria.Desulfuromonadales | High | 3.137 | 0.035 |
| Bacteria.Chloroflexi.Anaerolineae | High | 3.098 | 0.027 |
| Bacteria.Planctomycetes.Planctomycetia.Planctomycetales.Planctomycetaceae.Blastopirellula | High | 3.033 | 0.028 |
| Bacteria.Bacteroidetes.Cytophagia.Cytophagales.Cytophagaceae.Adhaeribacter | High | 2.980 | 0.046 |
| Bacteria.Verrucomicrobia.Verrucomicrobiae.Verrucomicrobiales.Verrucomicrobiaceae.Prosthecobacter | High | 2.973 | 0.008 |
| Bacteria.Proteobacteria.Deltaproteobacteria.Desulfuromonadales.Geobacteraceae | High | 2.959 | 0.035 |
| Bacteria.Actinobacteria.Actinobacteria.Actinomycetales.Cryptosporangiaceae | High | 2.956 | 0.027 |
| Bacteria.Proteobacteria.Deltaproteobacteria.Myxococcales.Cystobacteraceae | High | 2.942 | 0.003 |
| Bacteria.Proteobacteria.Alphaproteobacteria.Rhizobiales.Rhodobiaceae.Parvibaculum | High | 2.892 | 0.036 |
| Bacteria.Armatimonadetes.Chthonomonadetes | High | 2.887 | 0.015 |
| Bacteria.Chloroflexi.Caldilineae.Caldilineales | High | 2.885 | 0.016 |
| Bacteria.Proteobacteria.Alphaproteobacteria.Rhizobiales.Rhodobiaceae | High | 2.874 | 0.021 |
| Bacteria.Proteobacteria.Deltaproteobacteria.Desulfuromonadales.Geobacteraceae.Geobacter | High | 2.859 | 0.035 |
| Bacteria.Acidobacteria.Acidobacteria_Gp1.Granulicella | High | 2.859 | 0.027 |
| Bacteria.Chloroflexi.Caldilineae | High | 2.847 | 0.012 |
| Bacteria.Proteobacteria.Deltaproteobacteria.Myxococcales.Polyangiaceae.Chondromyces | High | 2.811 | 0.004 |
| Bacteria.Acidobacteria.Acidobacteria_Gp1.Gp1 | High | 2.801 | 0.049 |
| Bacteria.Chloroflexi.Anaerolineae.Anaerolineales.Anaerolineaceae | High | 2.753 | 0.039 |
| Bacteria.Chloroflexi.Anaerolineae.Anaerolineales | High | 2.720 | 0.039 |
| Bacteria.Armatimonadetes.Chthonomonadetes.Chthonomonadales | High | 2.641 | 0.045 |
| Bacteria.Actinobacteria.Actinobacteria.Actinomycetales.Streptosporangiaceae.Nonomuraea | High | 2.627 | 0.045 |
| Bacteria.Armatimonadetes.Chthonomonadetes.Chthonomonadales.Chthonomonadaceae | High | 2.625 | 0.035 |
| Bacteria.Bacteroidetes.Sphingobacteriia.Sphingobacteriales | Low | 3.898 | 0.009 |
| Bacteria.Bacteroidetes | Low | 3.888 | 0.009 |
| Bacteria.Bacteroidetes.Sphingobacteriia | Low | 3.850 | 0.005 |
| Bacteria.Proteobacteria.Gammaproteobacteria.Xanthomonadales.Xanthomonadaceae | Low | 3.841 | 0.046 |
| Bacteria.Bacteroidetes.Sphingobacteriia.Sphingobacteriales.Chitinophagaceae | Low | 3.724 | 0.027 |
| Bacteria.Proteobacteria.Alphaproteobacteria.Rhizobiales.Phyllobacteriaceae | Low | 3.573 | 0.036 |
| Bacteria.Proteobacteria.Alphaproteobacteria.Rhizobiales.Phyllobacteriaceae.Aminobacter | Low | 3.500 | 0.046 |
| Bacteria.Bacteroidetes.Flavobacteriia.Flavobacteriales.Flavobacteriaceae.Flavobacterium | Low | 3.435 | 0.036 |
| Bacteria.Bacteroidetes.Sphingobacteriia.Sphingobacteriales.Sphingobacteriaceae | Low | 3.404 | 0.021 |
| Bacteria.Proteobacteria.Alphaproteobacteria.Rhizobiales.Bradyrhizobiaceae | Low | 3.387 | 0.016 |
| Bacteria.Proteobacteria.Gammaproteobacteria.Xanthomonadales.Xanthomonadaceae.Arenimonas | Low | 3.348 | 0.012 |
| Bacteria.Proteobacteria.Alphaproteobacteria.Caulobacterales.Caulobacteraceae.Caulobacter | Low | 3.336 | 0.005 |
| Bacteria | Low | 3.313 | 0.021 |
| Bacteria.Proteobacteria.Alphaproteobacteria.Sneathiellales | Low | 3.018 | 0.027 |
| Bacteria.Verrucomicrobia.Spartobacteria.Terrimicrobium | Low | 2.989 | 0.012 |
| Bacteria.Bacteroidetes.Sphingobacteriia.Sphingobacteriales.Sphingobacteriaceae.Sphingobacterium | Low | 2.967 | 0.049 |
| Bacteria.Proteobacteria.Alphaproteobacteria.Sneathiellales.Sneathiellaceae | Low | 2.945 | 0.027 |
| Bacteria.Proteobacteria.Alphaproteobacteria.Rhizobiales.Hyphomicrobiaceae.Devosia | Low | 2.917 | 0.027 |
| Bacteria.Bacteroidetes.Sphingobacteriia.Sphingobacteriales.Chitinophagaceae.Ferruginibacter | Low | 2.891 | 0.036 |
| Bacteria.Actinobacteria.Actinobacteria.Actinomycetales.Sporichthyaceae.Sporichthya | Low | 2.815 | 0.012 |
| Bacteria.Proteobacteria.Alphaproteobacteria.Sneathiellales.Sneathiellaceae.Ferrovibrio | Low | 2.795 | 0.027 |
| Bacteria.Bacteroidetes.Cytophagia.Cytophagales.Chryseolinea | Low | 2.778 | 0.012 |
| Bacteria.Actinobacteria.Actinobacteria.Actinomycetales.Sporichthyaceae | Low | 2.762 | 0.016 |
| Bacteria.Proteobacteria.Gammaproteobacteria.Pseudomonadales.Moraxellaceae.Acinetobacter | Low | 2.703 | 0.021 |

Supplementary Table 5. Pearson correlation among strawberry plant traits in the pathogen trial infested with the soil-borne fungal pathogen *Verticillium dahliae*, including plant biomass, nutrient content, and microbial diversity in the rhizosphere (Shannon diversity Index)*.* Significant correlations are denoted by * (p<0.01) and ** (p<0.001)

|  | Biomass  (g plant^-1^) | Leaf N (%) | Leaf Ca  (%) | Leaf K  (%) | Leaf Mg (%) | Leaf P  (%) | Ca:Mg | Shannon Index |
| --- | --- | --- | --- | --- | --- | --- | --- | --- |
| Leaf N (%) | -0.606** |  |  |  |  |  |  |  |
| Leaf Ca (%) | 0.091 | -0.4* |  |  |  |  |  |  |
| Leaf K (%) | -0.041 | 0.029 | -0.186 |  |  |  |  |  |
| Leaf Mg (%) | -0.257 | -0.132 | 0.775 | -0.432 |  |  |  |  |
| Leaf P (%) | 0.161 | -0.181 | -0.019** | 0.320* | -0.316 |  |  |  |
| Ca:Mg | 0.470* | -0.546** | 0.709 | 0.182 | 0.111 | 0.338 |  |  |
| Shannon diversity Index | -0.358 | 0.209 | 0.368* | -0.163 | 0.510** | -0.029 | 0.009 |  |
| Plant mortality (%) | -0.128 | 0.563** | -0.595** | 0.225 | -0.531** | -0.033 | -0.373* | -0.343 |

Supplementary Table 6. Pearson correlation among strawberry plant traits in the pathogen trial inoculated with the soil-borne fungal pathogen *Macrophomina phaseolina*, including plant biomass, nutrient content and microbial diversity in the rhizosphere soil (Shannon diversity Index)*.* Significant correlations are denoted by * (p<0.01) and ** (p<0.001)

|  | Biomass  (g plant^-1^) | Leaf N  (%) | Leaf Ca (%) | Leaf K  (%) | Leaf Mg  (%) | Leaf P (%) | Ca:Mg | Shannon Index |
| --- | --- | --- | --- | --- | --- | --- | --- | --- |
| Leaf N (%) | -0.267 |  |  |  |  |  |  |  |
| Leaf Ca (%) | -0.157 | 0.031 |  |  |  |  |  |  |
| Leaf K (%) | 0.535** | 0.217 | -0.324* |  |  |  |  |  |
| Leaf Mg (%) | -0.266 | 0.255 | 0.844** | -0.353* |  |  |  |  |
| Leaf P (%) | 0.203 | 0.119 | 0.377* | 0.487** | 0.203 |  |  |  |
| Ca:Mg | 0.091 | -0.283 | 0.696** | -0.082 | 0.210 | 0.439** |  |  |
| Shannon diversity Index | -0.024 | -0.244 | -0.196 | -0.005 | -0.258 | 0.026 | -0.023 |  |
| Plant Mortality (%) | -0.075 | 0.065 | 0.195 | -0.238 | 0.080 | 0.093 | 0.242 | -0.101 |

Supplementary Table 7. Summary of the 16 cultivars used in each field trial. The cultivars showed different levels of resistance against the two soil borne pathogens *Verticilium dahliae* and *Macrophomina phaseolina*.

| *V. dahliae* trial | | | | *M. phaseolina* trial | | |
| --- | --- | --- | --- | --- | --- | --- |
| Cultivar | Code | | Breeding program | Cultivar | Code | Breeding program |
| BG 4.367 | | BG4 | Planasa | BG 4.367 | BG4 | Planasa |
| Festival | | FEL | University of Florida | Festival | FEL | University of Florida |
| Marquis | | MS | Driscoll’s | Marquis | MS | Driscoll’s |
| Petaluma | | PA | UC Davis | Petaluma | PA | UC Davis |
| Benicia | | BA | UC Davis | Albion | ABN | UC Davis |
| BG 1975 | | BG1 | Planasa | Del Rey | DR | Driscoll’s |
| Camino Real | | CR | UC Davis | El Dorado | ELD | Driscoll’s |
| Odessa | | OA | Driscoll’s | Grenada | GNA | UC Davis |
| San Andreas | | SA | UC Davis | Monterey | MOY | UC Davis |
| UC-12 | | UC | UC Davis | UC-J | UCJ | UC Davis |

*UC Davis: University of California Davis

Supplementary Figure 1. Non-metric multidimensional scaling (NMDS) ordinations based on the Bray-Curtis similarity of OTU based bacterial and archaeal community structure across four strawberry cultivars (BG4: blue, FEL: grey, MS: orange, PA: pink), grown in *Macrophomina phaseolina* (solid symbols), and *Verticilium dahliae* (open symbols) infested fields.


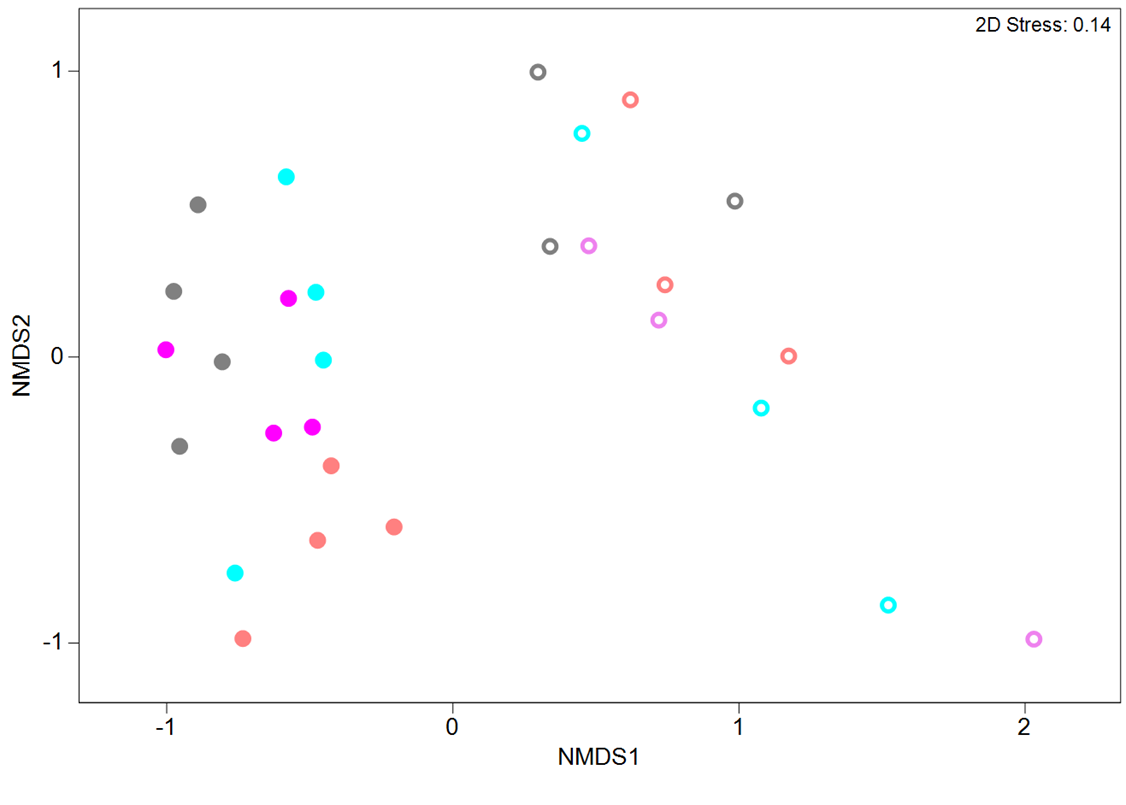


Supplementary Figure 2. Overview of the field set up showing the experimental and control plots, each containing 90 plots (left), plots and sampling scheme (right) followed in the *Verticilium dahliae* and *Macrophomina phaseolina* field trials. Four strawberry plants of the same cultivar were randomly selected among the healthy plants in the same 20-plant plot. Selected plants were flagged (see picture in the right), sampled by separating roots and shoots in each plant and combining the plants for one final shoot and one rhizosphere composite sample per plot. Additionally, one bulk soil sample was also collected from each plot. Aerial photography of the field was kindly provided by Dr. Bo Liu (California Polytechnic State University, San Luis Obispo).
